# *Cryptococcus neoforman*s rapidly invades the murine brain by sequential breaching of airway and endothelial tissues barriers, followed by engulfment by microglia

**DOI:** 10.1128/mbio.03078-23

**Published:** 2024-03-21

**Authors:** Vanessa I. Francis, Corin Liddle, Emma Camacho, Madhura Kulkarni, Samuel R. S. Junior, Jamie A. Harvey, Elizabeth R. Ballou, Darren D. Thomson, Gordon D. Brown, J. Marie Hardwick, Arturo Casadevall, Jonathan Witton, Carolina Coelho

**Affiliations:** 1 MRC Centre for Medical Mycology at University of Exeter, University of Exeter, Exeter, United Kingdom; 2 Faculty of Health and Life Sciences, University of Exeter, Exeter, United Kingdom; 3 Bioimaging Facility, University of Exeter, Exeter, United Kingdom; 4 Johns Hopkins Bloomberg School of Public Health, Baltimore, Maryland, USA; University of Florida College of Dentistry, Gainesville, Florida, USA

**Keywords:** microglia, *Cryptococcus neoformans*, blood-brain barrier

## Abstract

*Cryptococcus neoformans* causes lethal meningitis and accounts for approximately 10%–15% of AIDS-associated deaths worldwide. There are major gaps in our understanding of how this fungus invades the mammalian brain. To investigate the dynamics of *C. neoformans* tissue invasion, we mapped fungal localization and host cell interactions in infected brain, lung, and upper airways using mouse models of systemic and airway infection. To enable this, we developed an *in situ* imaging pipeline capable of measuring large volumes of tissue while preserving anatomical and cellular information by combining thick tissue sections, tissue clarification, and confocal imaging. We confirm high fungal burden in mouse upper airway after nasal inoculation. Yeast in turbinates were frequently titan cells, with faster kinetics than reported in mouse lungs. Importantly, we observed one instance of fungal cells enmeshed in lamina propria of the upper airways, suggesting penetration of airway mucosa as a possible route of tissue invasion and dissemination to the bloodstream. We extend previous literature positing bloodstream dissemination of *C. neoformans*, by finding viable fungi in the bloodstream of mice a few days after intranasal infection. As early as 24 h post systemic infection, the majority of *C. neoformans* cells traversed the blood-brain barrier, and were engulfed or in close proximity to microglia. Our work presents a new method for investigating microbial invasion, establishes that *C. neoformans* can breach multiple tissue barriers within the first days of infection, and demonstrates microglia as the first cells responding to *C. neoformans* invasion of the brain.

**IMPORTANCE:**

Cryptococcal meningitis causes 10%–15% of AIDS-associated deaths globally. Still, brain-specific immunity to cryptococci is a conundrum. By employing innovative imaging, this study reveals what occurs during the first days of infection in brain and in airways. We found that titan cells predominate in upper airways and that cryptococci breach the upper airway mucosa, which implies that, at least in mice, the upper airways are a site for fungal dissemination. This would signify that mucosal immunity of the upper airway needs to be better understood. Importantly, we also show that microglia, the brain-resident macrophages, are the first responders to infection, and microglia clusters are formed surrounding cryptococci. This study opens the field to detailed molecular investigations on airway immune response, how fungus traverses the blood-brain barrier, how microglia respond to infection, and ultimately how microglia monitor the blood-brain barrier to preserve brain function.

## INTRODUCTION

*Cryptococcus neoformans* has been designated by the World Health Organization as a critical priority pathogen that causes ~112,000 deaths per year including 19% of HIV-associated deaths (1). Healthy individuals acquire *C. neoformans* infections from environmental sources, and 56%–70% of healthy children ages 1–10 years have serum antibodies against *C. neoformans* proteins (2). Such early seropositivity suggests individuals frequently come into contact with *C. neoformans* and that infection in healthy individuals is either cleared or persists in a latent, asymptomatic form (3, 4). Dissemination from airways requires *C. neoformans* to cross a series of tissue barriers to exit the airways, enter the bloodstream, and cross the blood-brain barrier (BBB) where it causes meningoencephalitis (5). Thus, *C. neoformans* can rapidly cross tissue barriers to invade the mammalian brain, suggesting the existence of sophisticated invasion mechanisms that are not understood.

We previously reported that viable *C. neoformans* could be recovered from mouse brains as early as 3 h, and fungal burden up to 7 days after intranasal infection of mice (6). Intravital microscopy studies detected *C. neoformans* traversal from the lumen of capillaries to the mouse brain parenchyma within a few hours after systemic infection (7–9), consistent with *in vitro* models of endothelial tissue infection (10–13).

How *C. neoformans* crosses tissue barriers on initial airway infection to reside in the brain is still poorly defined. To efficiently identify the earliest sites of dissemination requires the capacity to observe and analyze rare, sparsely distributed invasion events. To achieve this goal, we implemented tissue clarification and decolorization, which remove lipids and certain pigments from tissues, resulting in a dramatic increase in tissue transparency, thus allowing high-content imaging of thick samples (14). This technique has great promise for studying host-pathogen interactions. This has been used to quantify *Aspergillus fumigatus* growth and association with host immune cells in whole lungs (15). This technique was also used to study cryptococcal infection-induced melanization of *Galleria mellonella*, an insect model of cryptococcal infection (16). Here, we combined tissue clarification with confocal microscopy to investigate early *C. neoformans* infection in mice airways and brain. In a model of intranasal infection, we present evidence for tissue barrier crossing in upper airways and lungs. We observed that within the first 24 h after intravenous infection, the majority of *C. neoformans* cells have traversed the BBB and are associated with brain-resident ionized calcium-binding adapter molecule 1 (Iba1^+^) macrophages. Our study is a key step toward defining the tissue routes and cellular interactions facilitating *C. neoformans* dissemination through mammalian hosts, and firmly implicates microglia as the primary brain immune cell responding to cryptococcal BBB traversal.

## RESULTS

### High-content imaging of *C. neoformans-*host interactions in multiple infected tissues

**Fig 1:**
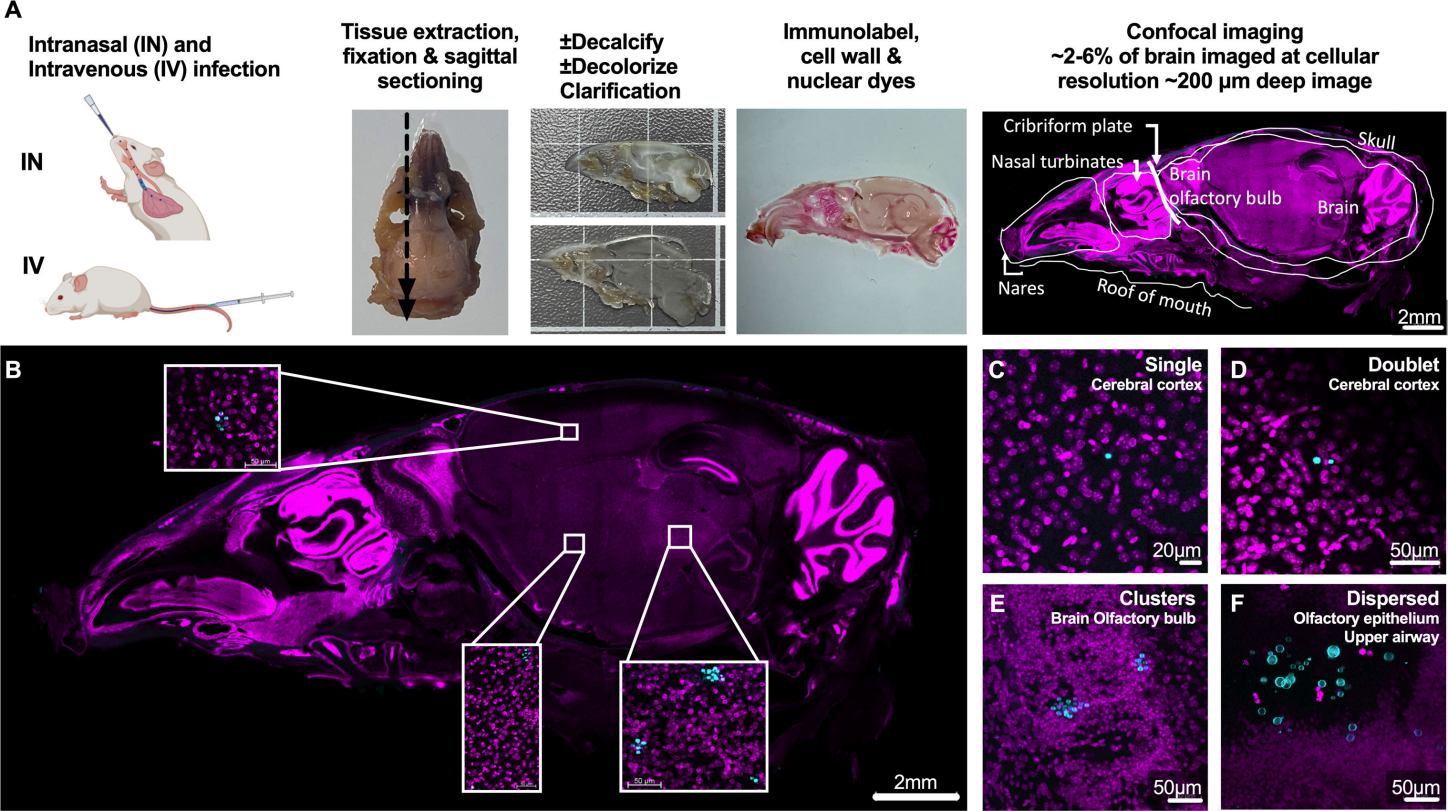
Workflow of high-content, subcellular resolution imaging of infected tissue slices. (**A**) Schematic of experimental protocol. Representative images of (**B**) a sagittal cut slice of infected skull, showing insets of location of *C. neoformans*, and (**C–F**) *C. neoformans* cells are observed in infected tissues as (**C**) single, (**D**) doublets, (**E**) clusters, and (**F**) dispersed. Images from skull of C57bl/6 male infected with 5 × 10^5^ H99E for 24 h, via intravenous (**B–E**) or intranasal infection (**F**). Brain and skulls were cut in sagittal sections, lungs into lobes or into coronal slices. Fungi were identified by cell wall staining with CFW (50 µg/mL, cyan) or specific antibodies (see Materials and Methods), and tissues counterstained with different nuclear dyes (magenta), propidium iodide (PI), Helix NP Blue, and Helix NP Green, depending on other fluorophores used. Shown are maximum intensity projections with a depths of (**C**) 36 µm (×5 z-steps), (**D**) 63 µm (×8 z-steps), (**E**) 105 µm (×36 z-steps), and (**F**) 109.47 µm (x11 z-steps). Note that the third panel in panel A is magnified in panel B and is the same section image as Fig. 6A. Scale bars in panels (see also Video S1).

To map early steps of host invasion by *C. neoformans in vivo*, we combined mouse infections, tissue clarification, and high-content imaging (workflow illustrated in Fig. 1). We note that in most tissue clearing work, perfusion is performed to reduce highly pigmented hemoglobin. We chose to not perform perfusion to avoid removal of *C. neoformans* yeast from the bloodstream. This was replaced with a decolorization step which degrades hemoglobin in the blood and other pigments to improve light penetration (17). Following infection with *C. neoformans*, mice were sacrificed at 1–7 days, and the skull and lungs were collected and fixed. Skulls were decalcified, cut into thick sagittal tissue sections (10–13 per mouse), and clarified using X-CLARITY. These sections provided an optimal balance between preservation of anatomical context, efficiency of data collection, and sampling capacity. Lungs were also collected, clarified, decolorized, and imaged whole, or in two to three coronal or axial sections. This was followed by staining for fungal cells with calcofluor white (CFW), for host cell nuclei with nuclear dyes, which serves as anatomical landmarks, and in some experiments for host immune cells using immunolabeling. In all, at least 200 µm depth of cleared skulls with 9 µm z-axial spacing per frame was analyzed, corresponding to ~4% of the brain volume (Fig. 1, see Materials and Methods). CFW dye can have background staining, including bone structures and debris of unknown nature. To control for this background, we imaged CFW-stained tissues from uninfected animals (Fig. S1) and noted CFW-stained debris did not have characteristic fungal morphology. Fungal cells were detected and confirmed via manual verification of characteristic cryptococcal morphology (Fig. 1, bottom panel, and Video S1). In summary, we developed a robust tissue clearing and imaging method that can be easily adopted to characterize pathogen invasion in rodent models.

### Quantification of fungal titan cells *in situ*

**Fig 2:**
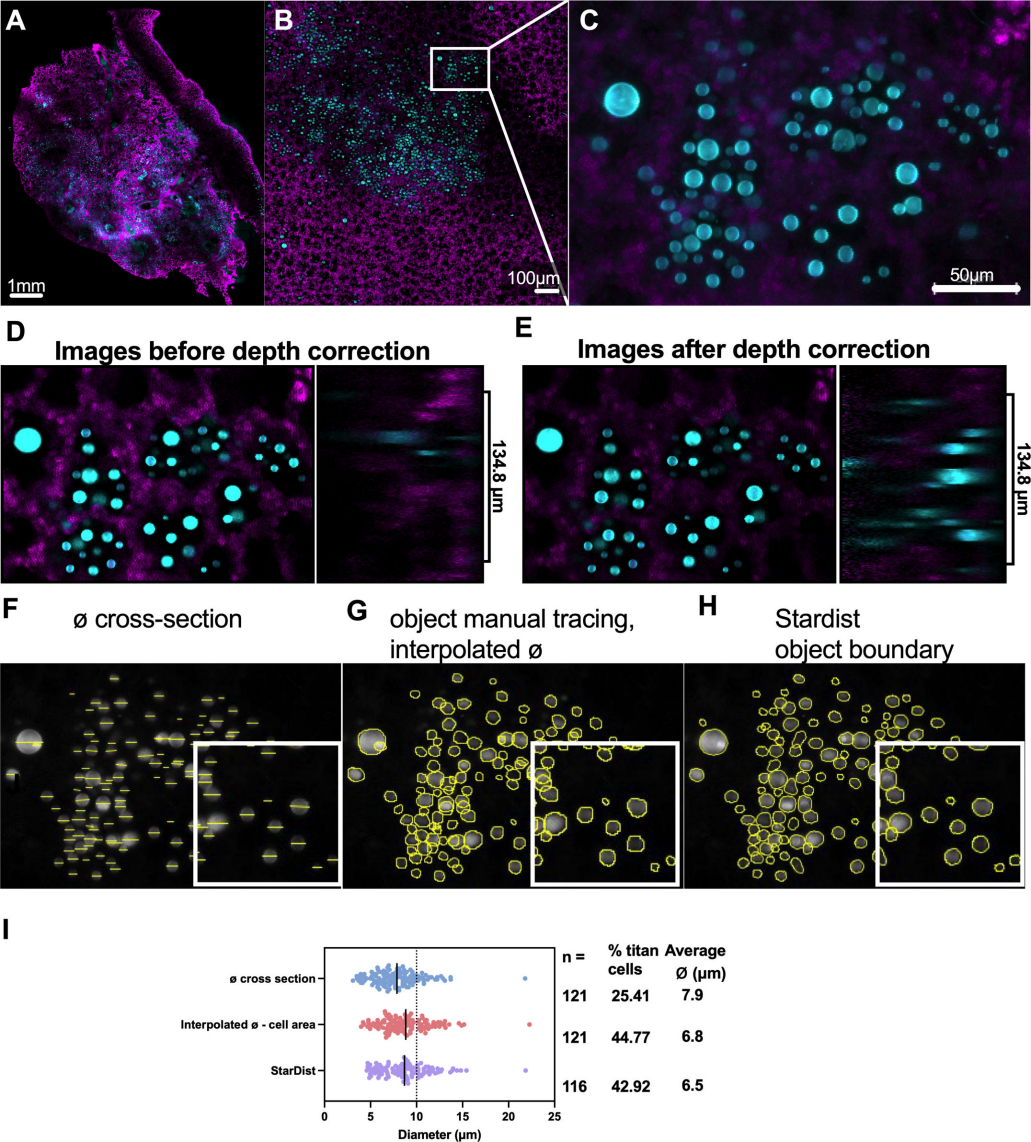
Pipeline to detect cryptococcal cell size distribution in infected tissues. Map of infected lung showing CFW-stained cryptococcal cells and PI-stained nuclei of lung cells, with consecutive magnifications from tissue to subcellular resolution. Shown are several steps in image processing and analysis that allowed measurements of fungal cell morphology *in situ*. Different methods were compared, including the standard method of direct diameter (∅ cross-section) versus an object boundary method which allow automated detection (StarDist). (**A**) Single plane tiling map. (**B**) Magnification and (**C**) Region of Interest (ROI) used for quantification. (**D**) Depth of tissue results in decreased signal intensity. Diminishing CFW signal in YZ projection (2.01 µm x 68 z-step) in a single XY single plane ROI, and (**E**) which is corrected by depth correction normalization function in ImageJ and improves signal uniformity. Fungal size (diameter) was measured in this ROI via three methods: (**F**) manually cross-section (ø cross-section), (**G**) manual object tracing, followed by area determination, and interpolation of diameter (interpolated ø), and (**H**) automated analysis using ImageJ StarDist macro, which uses thresholding to define object boundaries, followed by area and diameter calculation (StarDist). (**J**) Comparison of all three methods used to measure cryptococci size. *n*, total number of cells detected, cells >10 µm are classified as titan cells (dashed line). Images (**B–I**) from lung of C57bl/6J male mice, 5 dpi intranasal with 5 × 10^7^ *C. neoformans ste50*Δ, a strain with virulence comparable to KN99 parental wild-type. Lungs stained with PI (magenta) and CFW (cyan). Data from one mouse. Images correspond to (**B–E**) extended depth of field image, 281.46 µm by 185.98 µm, with 134.8 µm depth with 2.01 µm × 68 z-steps. Scale bar in images, except panels F, G, and H, which represent 281.46 µm by 185.98 µm.

Although *C. neoformans* titan cells (defined as having cell body >10 µm diameter [18]) are important *in vivo*, they have not previously been characterized *in situ*, which was made possible with our new techniques. Volumetric imaging of fungal cells in mouse lung tissue (Fig. 2A through C) revealed a wide range of cryptococcal cell sizes. To measure fungal size accurately, correction for loss of light intensity over the depth of tissues was performed (illustrated in Fig. 2D vs E). We measured fungal size in these images via the standard method of manually measuring cells in cross-sections (Fig. 2F). We also used semi-automated analysis via StarDist from ImageJ; this is based on intensity thresholding fluorescent signals to define an object’s boundary. From these data, the area of the object can be used to calculate the diameter of the object. We also decided to manually trace the circumference boundary of fungi to directly compare to StarDist object boundary tracing (Fig. 2G vs H ; see also Materials and Methods, and Supplemental Results and Methods). Comparison of all three methods showed that manual boundary tracing and StarDist yielded similar results, supporting the accuracy of the StarDist approach. Measuring diameter via cross-section showed some discrepancies from both methods of boundary tracing. Consistent with previous work, ~40% of cryptococci, as determined by StarDist in the lung, were titan cells (Fig. 2I). We further confirmed our method was accurate by testing conditions in which titan cells are rare (Fig. S2 and Supplemental Results and Methods). Testing whether tissue processing affected fungal cell size (Fig. S2 and Supplemental Results and Methods) showed fungal cell size was not affected in lung tissue; in certain conditions, a maximum increase of 1.13-fold in mean diameter may occur (see Supplemental Results and Methods for additional details). Thus, our imaging and analysis pipelines readily detect differences in fungal size *in situ*. Our method can also be harnessed to obtain greater depth of imaging in widefield microscope compared to non-clarified samples. Using widefield fluorescence microscopy, we obtained z-stacks with high-quality images at >82 µm depth in a single tissue slice (see details in Fig. S3, and supplemental Results and Methods).

### Abundant *C. neoformans* titan cells are present in upper airways by 1 day post infection

**Fig 3:**
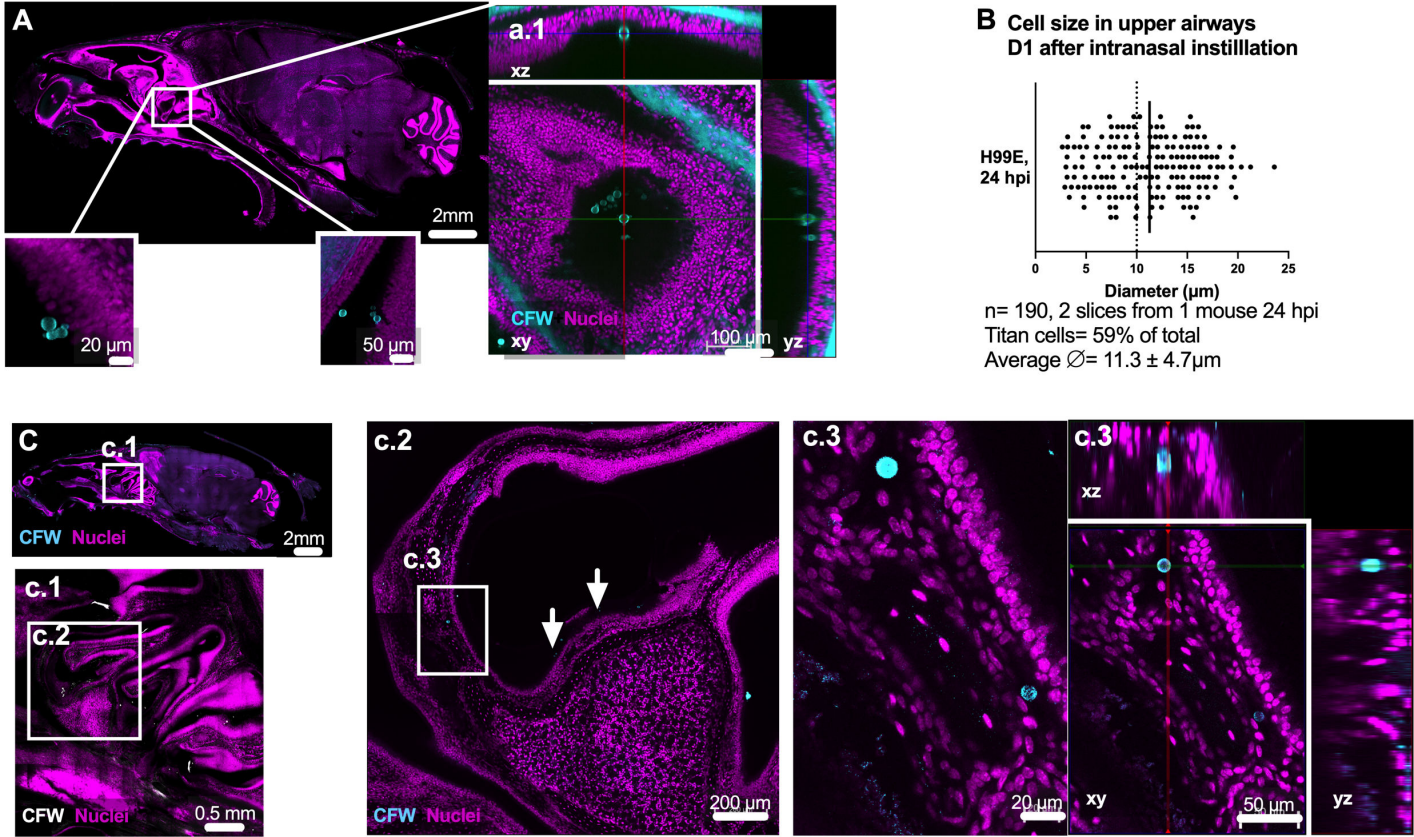
Presence of abundant titan cells in mouse airways 24 h after intranasal instillation of yeast. *C. neoformans* yeast and titan cells are abundant in upper airways within turbinates, closely apposed to, and invading lamina propria of the olfactory mucosa. (**A**) Skull slice, showing several instances of cryptococci apposed to mucosa with region of interest (ROI) magnified in panel a.1 (see Video S1 to visualize YZ). (**B**) Titan cells are abundant in olfactory mucosa, as early as 24 hpi after intranasal inoculation of H99E 5 × 10^5^ CFU. Cryptococci size measured using StarDist; (**C**) cryptococci can be observed throughout the upper airway and invading mucosa, with (c.1) highlighting location of fungal cells, and (c.2) cryptococci within turbinates (white arrows) and (c.2–3) enmeshed in lamina propria, below mucosal layer; fungi cell body diameters are, respectively, 12.98 µm (top) and 9.85 µm (bottom) measured by StarDist. Images shown are (**A**) skull single plane, (a.1) xy single planes with xyz projection (right panels). (**B**) Quantification of cryptococci size from two skull slices from the same animal imaged with a depth of 225 and 208 µm. (**C**) Maximum projection of skull slice (same animal as panel A, 2 × 27 µm z-step). (c.1) Max projection 225 µm, 26 × 9 µm z-step; (c.2) 6 µm max projection (2 × 6 µm z-step); (c.3) 84 µm (15 × 6 µm z-step) and orthogonal view. Data from one C57bl/6J mouse, confirmed in two additional CX3Cr1^GFP/+^ mice, with one to two slices imaged in each mouse. Sagittal slices corresponding to Allen Brain map slices (**A**) 15–19 and (**C**) 11–15. Scale bar indicated in images. GFP, green fluorescent protein.

**Fig 4:**
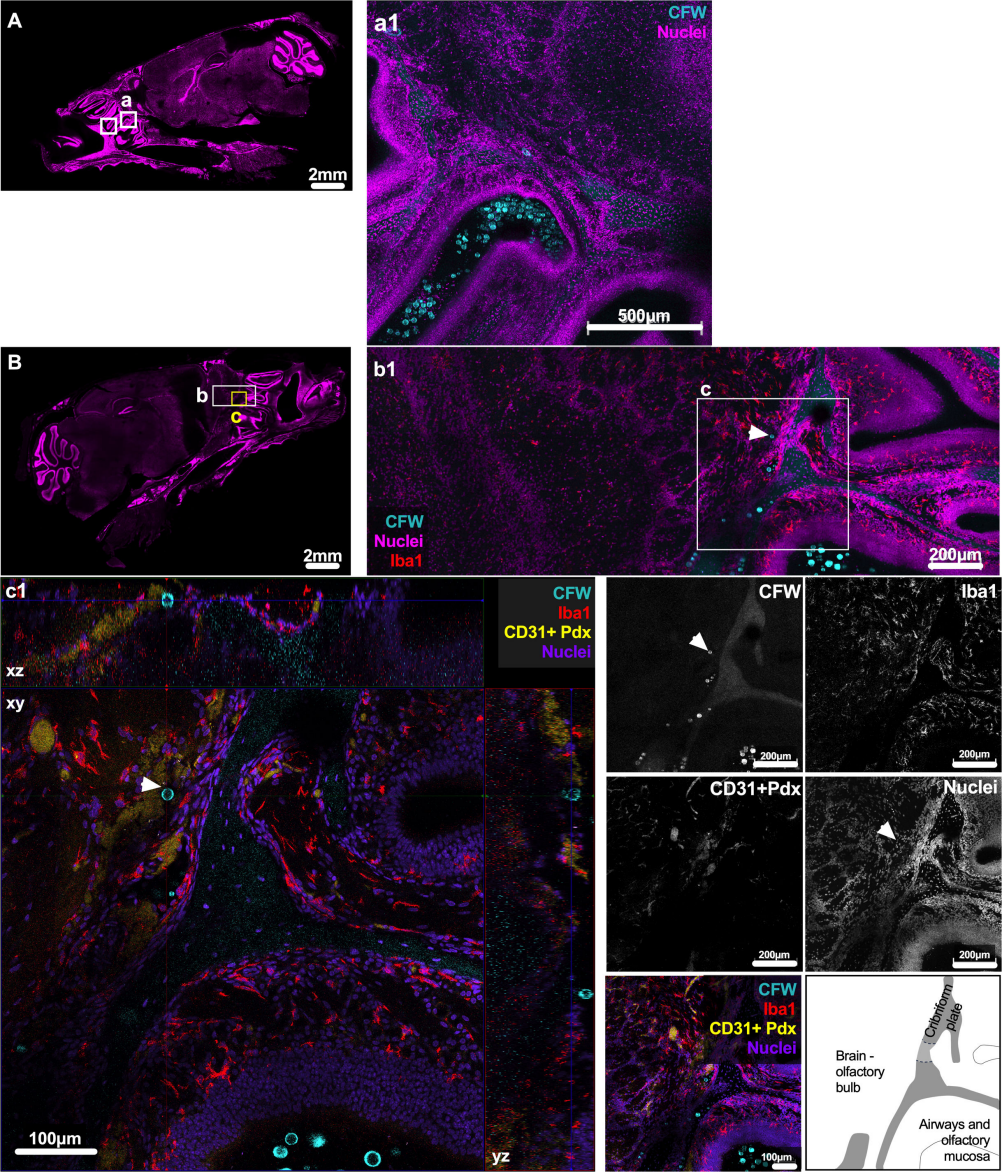
Presence of titan cells in mouse brain at 7 days post-intranasal infection, located near the cribriform plate in mouse brain, and persistence of fungi in mouse upper airways. *C. neoformans* fungi, including titan cells, are present in the brain olfactory bulb, above the cribriform plate at 7 dpi. Fungi continue to be abundant in upper airways and found attached to olfactory mucosa. (**A and B**) *C. neoformans* fungi found in upper airways and ethmoturbinates (boxes). (**a1**) Abundant cryptococci, including titan cells in upper airways, confirmed in 3/3 mice. (**b1 and c1**) *C. neoformans* cells in brain above cribriform plate, as well as yeast in upper airway just below cribriform plate, data from 1/3 mice imaged. (**c1**) Titan cell located in brain olfactory bulb (white arrow, 17.79 µm diameter measured by StarDist), above the cribriform plate. Images shown are (**A and B**) single plane of skull sections, (**a1**) maximum intensity projection comprising 68 µm (4 µm × 18 z-steps) deep, (**b1 and c1**) xyz projection with depth of 145.51 µm (2.35 µm × 63 z-steps), and maximum intensity projections of same area with a depth of 28.16 µm (2.35 µm × 13 z-steps). Images from one C57bl/6J mouse, and two additional CX3Cr1^GFP/+^ mice, with 3–10 slices imaged in each mouse. Slices shown correspond to Allen Brain map 15–19. For panels A–C, fungi cell wall CFW in cyan, nuclei in magenta, Iba1 in red, CD31 + Pdx in yellow, with single color images in grayscale. Scale bar indicated in images.

We previously showed that after intranasal inoculation, fungi are detectable in the upper airways as early as 24 hpi, with some fungal cells already forming titan cells (6). For a more quantitative analysis, our high-content imaging approach was applied to skulls of mice infected via the intranasal route at 24 hpi and 7 dpi (6). Imaging of sagittal slices taken from infected skulls, using the same dose and fungal strain as previous studies, showed cryptococci distributed throughout the upper airway turbinates at both 24 hpi and 7 dpi (Fig. 3 and 4). At 24 hpi, yeast cells were adhered to the olfactory mucosa in nasal turbinates, including the superior turbinates (ethmoturbinates, most distal from nostrils—illustrated in Fig. 3, and Video S2), indicating capacity of yeast cells to overcome the first anatomical filtering barriers of the airways and to adhere to epithelial surfaces in these distal regions (Fig. 3, panel a1). Remarkably, >50% of cryptococci in airways at 24 hpi were titan cells (Fig. 3B), which indicates for the first time that the environment in airway turbinates is a strong inducer of titan cell formation. We also surprisingly observed a large (≥10 µm) cryptococcal cell (Fig. 3C) located in the lamina propria below the olfactory mucosa at 24 hpi (Fig. 3C, panel c.3), suggesting invasion of airway mucosa, which had not been described before. At 7 dpi, we continued to observe abundant fungi in upper airways, including a high proportion of titan cells (Fig. 4A, panel a1).

### *C. neoformans* is present in mouse brain parenchyma 7 days after intranasal infection

Our previous work showed that viable cryptococci could be found in mouse brains as soon as 3–24 h after intranasal infection (6), and could persist for the duration of infection (6, 19). To characterize brain invasion dynamics, clarified skulls were co-immunolabeled for blood vessels using two abundant endothelial markers, CD31 and podocalyxin (CD31 + Pdx) (20). These sections were also immunolabeled for Iba1, a marker of microglia (brain-resident macrophages) (21, 22). We confirmed specificity of this staining via single color controls (Fig. S4). We also confirmed that in these sections, Iba1 staining co-localized with green fluorescent protein (GFP) expression in CX3Cr1^GFP/+^ mouse brains, as CX3Cr1^GFP/+^ mice are frequently used to label and identify microglia in imaging and flow studies (Fig. S5). Both Iba1 and GGFP staining showed the characteristic microglia morphology (Fig. S4 and S5). However, we note that these two markers are markers of several microglia subsets, and do not distinguish between parenchymal microglia versus recently described border-associated macrophages (23, 24). We first confirmed that at 7 dpi in an intranasal infection, a fungal burden ranging from 21 to 260 CFU could be found in brains of mice (*n* = 3 mice, 3/3 mice positive, data not shown), and reasoned our tool may be powerful enough to locate fungi in this model. Imaging and inspection of brain parenchyma from mice culled 7 days after intranasal infection showed one instance of cryptococci in the brain at 7 dpi, out of three animals imaged (Fig. 4B); this cryptococci was located in the olfactory bulb above the cribriform plate. This cryptococcal cell was not associated with microglia, as shown by staining of microglia Iba1^+^ cells (Fig. 4B, panel c.1). Consistent with our previous observations (6), cryptococci have already disseminated to the murine brain at 7 dpi, albeit at low frequency.

### *C. neoformans* is present in the bloodstream as free yeast at 3 and 7 days after intranasal infection

**Fig 5:**
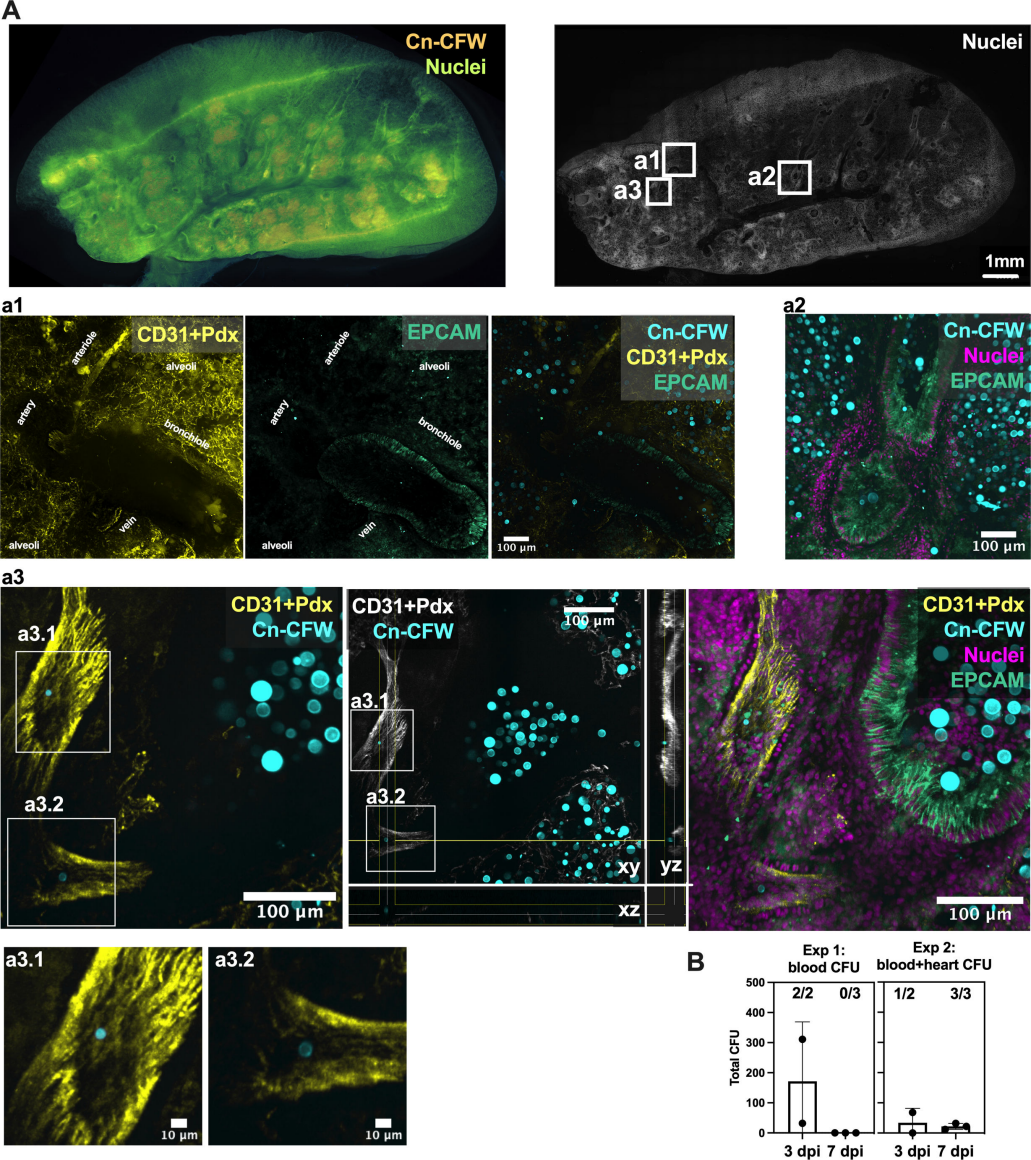
Cryptococci access bloodstream at 3 and 7 days after intranasal infection. (**A**) Lung section map allows visualization of fungi in lungs (left) followed by confocal imaging (right) showing cryptococci situated in alveoli (**A, a1**). A significant portion of cryptococci found in EpCAM^+^ airways (**a2–a3**), particularly in bronchioles and terminal bronchioli, and rare instances of cryptococci found in blood vessels (**a3**), identified by CD31 + Pdx endothelium staining. (**B**) Viable fungi can be found in the bloodstream after intranasal infection at 3 dpi (cardiac bleed) and 7 dpi (cardiac bleed + heart homogenates). Numbers above bars indicate [mice with CFU in tissue/total mice tested]. Images are maximum projections from (**A**) 18 × 25 µm z-step, (**a1**) 23 × 3 µm z-step, (**a2**) 14 × 13 µm z-step, (**a3**) 12 × 13 µm z-step, and xyz projection in middle panel. Data from (**A**) CX3Cr1^GFP/+^, data from 1/1 mouse, and (**B**) C57bl/6J and CX3Cr1^GFP/+^ mice, all intranasally infected with 5 × 10^5^ CFU of mCardinal H99. Data points represent individual mice, each experiment, *n* = 2 at 3 dpi and *n* = 3 at 7 dpi. For panel A, colors are CFW in orange and nuclei in green-blue, and represented with a transparency overlay. For panels a1–a3, colors are CFW in cyan, nuclei in magenta, CD31 + Pdx in yellow (grayscale in xyz projection), EPCAM in green (Sea green). Scale bar in images.

Several studies have shown viable cryptococci in the blood of infected mice as early as 24 hpi and up to 7 dpi in spleen and lymph nodes, which was interpreted to suggest that cryptococci disseminate from the lung to the brain via the lymph nodes, carried by antigen-presenting cells in a Trojan-horse mechanism (25). However, other works showed that free cryptococci can adhere and traverse human brain endothelial cells (26), become internalized by mouse brain endothelial cells, without the presence of phagocytes (27), and adhere to human lung epithelial-derived A549 cells (9, 28) and other airway immortalized cells (29), which would mean free yeast could cross alveoli and/or bronchioli. Thus, we attempted to observe whether we could observe (i) direct association of cryptococci with alveoli and (ii) phagocyte-associated or free yeast in bloodstream. Since lungs contain a high percentage of total blood volume, we could investigate both these questions by imaging infected mouse lungs. Lung from one animal was harvested 7 days after intranasal infection (Fig. 5), stained for fungi, and counterstained with CD31 + Pdx to label blood vessels, and with epithelial cell adhesion molecule (EpCAM) to label airway epithelium. As expected, imaging of lungs revealed abundant cryptococci distributed through alveoli (Fig. 5A) and other larger airways (Fig. 5a2). We did not detect direct crossing through alveoli or bronchiole, and with our limited sampling, it remains inconclusive as to whether this alveolar crossing occurs. However, we detected three instances of cryptococci located in major blood vessels, identified via CD31 + Pdx staining (Fig. 5a3). We did not observe host nuclei adjacent to yeast cells, which suggests that yeast in the blood stream are free yeast, and not located within phagocytes as posited by the Trojan-horse mechanism (25, 30). To confirm viable cryptococci can be found in the murine bloodstream in the first few days after intranasal infection, we additionally quantified CFU in blood extracted via intracardiac puncture (Fig. 5B). In a second experiment, we reasoned that part of the blood could remain inside the heart cavities even after intracardiac puncture, and thus, we performed intracardiac puncture followed by homogenizing the hearts and quantified CFU from both bleed and homogenized hearts. In both experiments, we confirmed cryptococci can be found in the bloodstream as early as 3 days post intranasal infection and up to 7 dpi, in agreement with previous work (25). We also confirmed viable fungi in lymph nodes and thymus (data not shown), as reported before (31). Taken together, these data show cryptococci in the bloodstream during early stages of intranasal infection, which would signify early seeding of brain after exposure to cryptococci. These data are also supportive of cryptococci in the bloodstream occurring as free yeast.

### Within the 24 h post systemic infection, *C. neoformans* is distributed through the brain, has crossed BBB, and associates with Iba1^+^microglia

**Fig 6:**
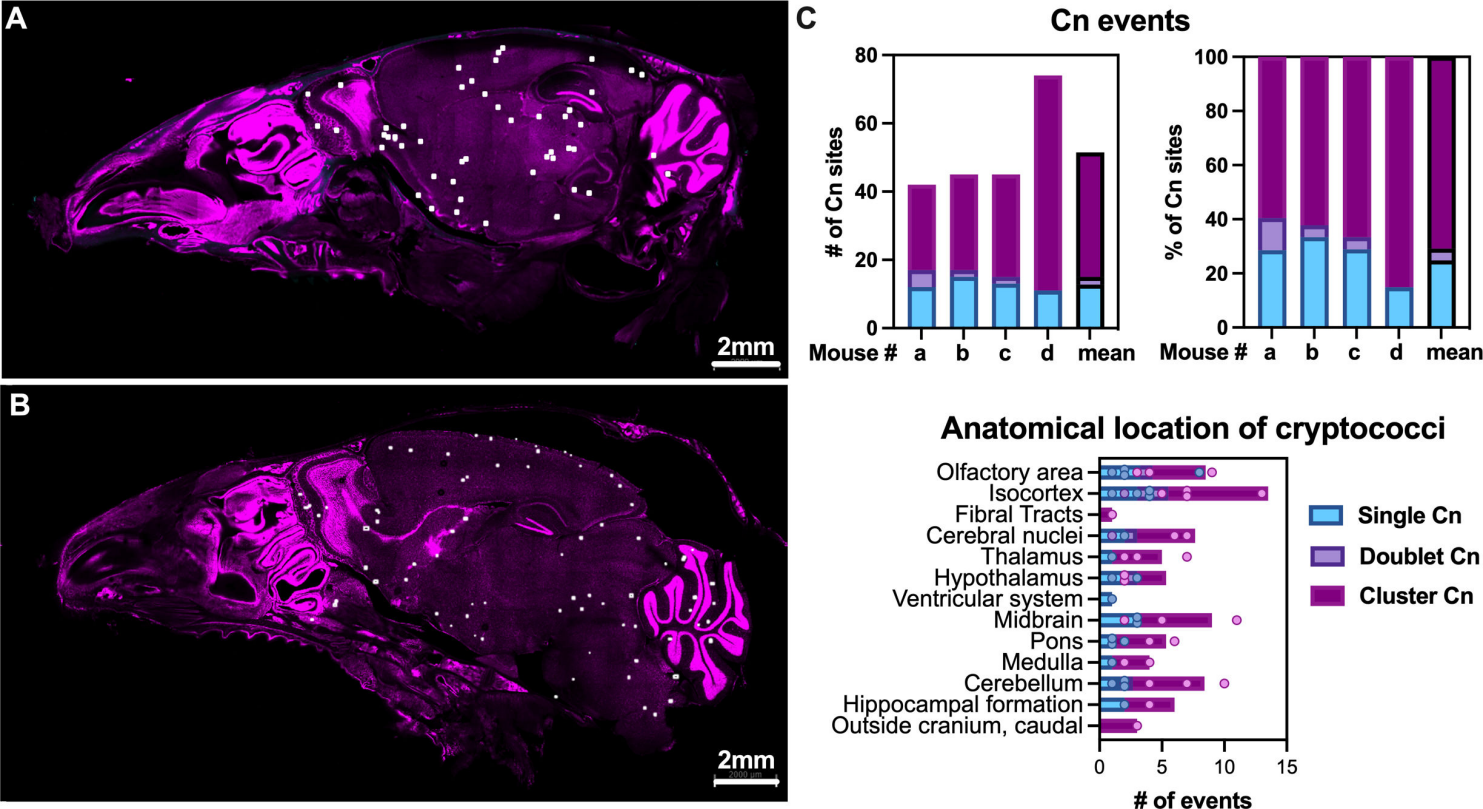
*C. neoformans* localization in brain after intravenous infection shows a dispersed pattern, consistent with bloodstream dissemination and passive arrest in capillaries.*C. neoformans* locations dispersed through the skull, most frequently found as clusters of >2 fungi, indicating either multiple cells traversing at the same location or that fungal cells are already replicating in tissue. (**A and B**) Representative sagittal sections of skulls from two C57bl/6J mice, with white dots indicating locations of *C. neoformans*. (**C**) Quantification of single, doublets, and clusters (>2 fungi, representative images in Fig. 1). Images shown are from two males C57bl/6, sagittal cuts corresponding to slices (**A**) 8–14 and (**B**) 13–19 of Allen Brain Atlas. (**C**) Graphs quantify four mice, two males C57bl/6 with 225-μm-thick sagittal section (26 × 9 µm z-steps), and two female CX3Cr1^GFP/+^ 140–150-μm-thick (5 × 35 µm z-steps, 6 × 30 µm z-steps), 1 day after tail vein i.v. with 5 × 10^5^ CFU of H99E and mCardinal strain, respectively. Top graphs show individual mice (labeled a through d; mean is also shown). Bottom graphs show each mouse as individual dots and bars represent mean of all mice. CFW in cyan, nuclei in magenta. Scale bar in images.

**Fig 7:**
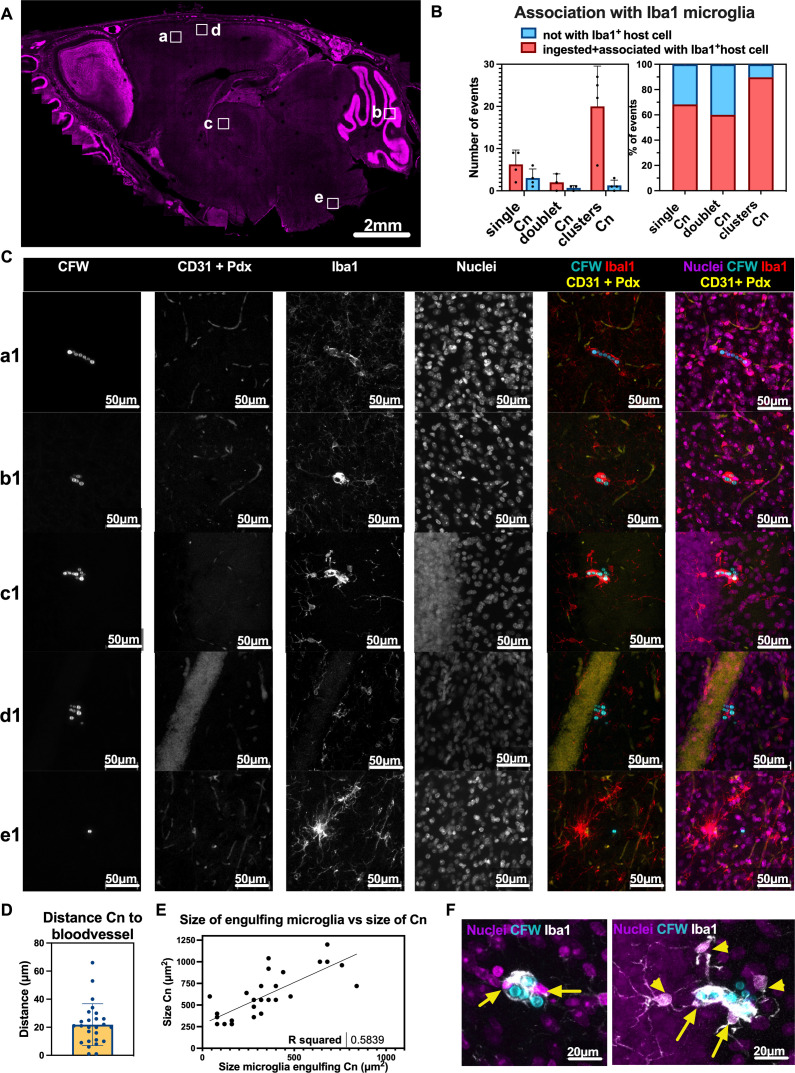
Traversal of the BBB by *C. neoformans* (Cn) leads to association with Iba1^+^ cells within 24 hpi. Association of fungal cells with Iba1^+^ cells, which may include brain-resident microglia and border-associated microglia in mouse brains, as early as 24 hpi. (**A**) Representative image of a skull with location of *C. neoformans*. (**B**) Quantification of microglia association with *C. neoformans*. (**C**) Representative images a1–e1 of cryptococci clusters associated with Iba1^+^ cells, and in proximity to CD31 + Pdx^+^ blood vessels, with panel e1 showing one cryptococci not associated with Iba1^+^ cells. (**D**) Distance of cryptococci to closest blood vessel, stained with CD31 + Pdx. (**E**) Area occupied by microglia increases with size of cryptococci cluster with (**F**) showing magnifications of b1 and c1, respectively, to show multiple nuclei of microglia (yellow arrows) and touching processes of neighboring microglia (yellow arrowheads). Images are maximum projections of (**A**) 140 µm (5 × 35 µm z-steps), corresponding to Allen Brain map sagittal sections 13–18; (**a1**) 75 µm (76 × 1 µm z-steps); (**b1**) 45 µm (46 × 1 µm z-steps); (**c1**) 81 µm (82 × 1 µm z-steps); (**d1**) 74 µm (75 × 1 µm z-steps); (**e1**) 69 µm (70 × 1 µm z-steps) (see Fig. S5 for representative images from C57bl/6J mice, and Fig. S6 and S7 for xyz projections of panels b1 and c1). Data from (**B**) *n* = 4 mice, data points correspond to individual mice, one skull section analyzed per mouse, 24 hpi i.v. infection with 5 × 10^5^ of strain mCardinal and H99E in two CX3Cr1^GFP/+^ female mice and two C57bl/6J male, respectively. For (**D and E**), data points represent each cryptococci cluster, data from two mice from randomly selected images, *n* = 26 cryptococci clusters in total. For panels A–C, fungi cell wall CFW in cyan, nuclei in magenta, Iba1 in red, CD31 + Pdx in yellow, with single colors in grayscale. For panel F, CFW in cyan, nuclei in magenta, Iba1 in grayscale. Scale bars in images.

To understand the events associated with *C. neoformans* invasion of the brain, we switched to an intravenous infection route that bypasses the airways and initiates more rapid—and therefore experimentally tractable—brain invasion in mice. Our finding of free cryptococci in blood after intranasal inoculation supports intravenous injection as a valid experimental model. To enable comparison of different *C. neoformans* strains, we infected CX3Cr1^GFP/+^ mice (C57Bl/6J background) and also C57bl/6J mice with fungal strain H99E or mCardinal-KN99α (mCardinal, data pooled in Fig. 6 and 7, Fig. S6 to S8, with *n* = 4 mice imaged in total). At 24 hpi, both *C. neoformans* strains were abundant and diffusely distributed throughout the brain (mapped in Fig. 6A and B), consistent with dissemination through the bloodstream (25). We found >74% (CI: 65%–84%) of cryptococci occurred in clusters (>2 yeast) instead of singlet or doublet cells (Fig. 6B). At 24 hpi in brain, cryptococci ranged from ~4 to 6 µm in diameter, and we did not detect titan cells nor fungal cells smaller than 3 µm in diameter in the brain parenchyma (see representative examples in Fig. 1C through F, in Fig. 7, and Fig. S6 to S8), in contrast to rapid induction of titan cells in upper airways, as shown in intranasal infections (Fig. 3).

To determine whether *C. neoformans* was present in brain blood vessels only or had crossed the blood vessels into the parenchyma at 24 hpi, we co-immunolabeled brain sections with Iba1 to label microglia and CD31 + Pdx to label the vascular endothelium. We mapped fungi in brain tissue, followed by higher-resolution imaging to quantify association with Iba1 microglia and with blood vessels (Fig. 7). We observed that the majority of cryptococci (80%) were fully or partially encased by Iba1^+^ cells (Fig. 7A through C, with representative examples in panels a1–e1 , Fig. S7 and S8, and Video S3a through d). Most cryptococci were adjacent to CD31 + Pdx vessels (Fig. 7C), with a mean distance to the closest blood vessel of 21.8 µm (CI: 15.8–27.9 μm). Only 20% of fungi were not associated with Iba1^+^ cells (example in panel e1 in Fig. 7C), and these may reside in the brain parenchyma associated with host cells such as astrocytes as reported by others (32, 33) (not labeled in our experiments) or as freely proliferating yeast in the perivascular and parenchymal space, as observed previously (34–36). We did not detect recruitment of peripheral phagocytes into the brain since GFP^+^,Iba1^-^ cells were not observed in the brain parenchyma of CX3Cr1^GFP/+^ mice, which is consistent with a >14-day delay in recruitment of peripheral immune cells to the brain in a systemic model of infection (37).

Observation of microglia morphology showed microglia cells were larger following engulfment of larger cryptococci (Fig. 7E), and we noted the presence of multiple host nuclei in the Iba1^+^ cluster surrounding *C. neoformans* (Fig. 7F). Inspection of the morphology of fungi-associated Iba1^+^ cells indicated these microglia assumed an amoeboid-like morphology, with fewer ramified processes (38) (Fig. 7C and F). Instances of neighboring microglia (Fig. 7F) extending processes toward cryptococci-containing microglia were noted, suggesting communication from infected microglia to neighboring microglia (38). The observed enlargement of Iba1^+^ microglia may occur by migration and fusion of neighboring microglia upon infectious stimuli; alternatively, microglia in response to inflammatory stimuli *in vitro* may become multinucleated due to cell proliferation with failed cytokinesis (39). We did not observe nuclear morphology suggestive of active proliferation by microglia, but our previous work showed that *in vitro* phagocytes and *in vivo* alveolar macrophages proliferate in response to cryptococcal infection (40, 41). These aspects will be dissected in future studies.

**Fig 8:**
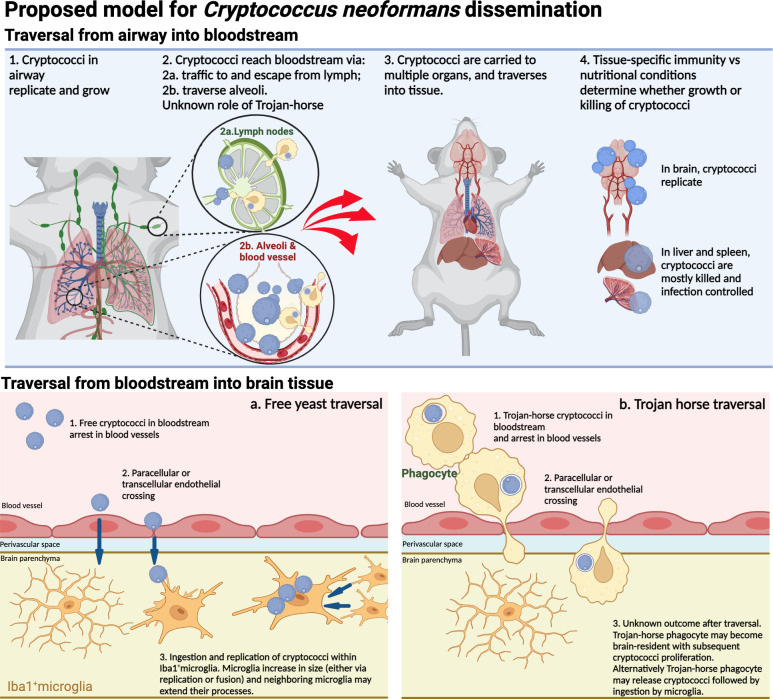
*Cryptococcus neoformans* traversal of barriers culminating in brain parenchyma invasion. Illustration shows proposed mechanisms currently supported by experimental evidence; relative predominance of both mechanism remains to be elucidated. It is also possible that there is a synergy between these mechanisms, with free yeasts traversal at certain sites and Trojan-horse in other sites. Iba1^+^ microglia may be brain-resident microglia or border-associated macrophages. Molecular mechanisms facilitating traversal of BBB are dependent on fungal metalloprotease Mpr1 (not depicted), and other interactions which are largely still to be elucidated. Figure created in Biorender.

Overall, our data show that within 24 h of systemic murine infection, *C. neoformans* crosses the BBB within the first hours after arresting in brain capillaries. Because the majority of cryptococci are in clusters, we propose two possible scenarios: (i) traversed cryptococci start to proliferate in the first 24 hpi, and possibly very soon after traversal of the BBB or (ii) cryptococci traversal creates a transient breach in the BBB, which can be exploited by subsequent cryptococci arrested at the same capillary site. In either case, soon after, traversal cryptococci in parenchyma would be phagocytosed by brain-resident microglia, prior to recruitment of peripheral monocytes (summarized in Fig. 8). Microglia may respond by enlarging and may show multiple nuclei when interacting with multiple cryptococci.

## DISCUSSION

In this work, we harnessed clarified tissue sections to image host-pathogen interactions in tissues at subcellular resolution. Thick sections (200 µm imaged) provided a substantial improvement step change in analyzing cryptococcal-tissue interactions and is easily adaptable to a wide range of microscopes. Thus, the imaging and analysis pipelines we established may be very easily adapted in other laboratories. The first challenge associated with this technique is reduced throughput, given long imaging times, long(er) post-acquisition processing, and size of data sets >100 Gb generated. A second challenge is that some antigens do not survive clarification, and thus some well-established antibodies cannot be used. A third challenge is to establish markers with a signal:noise ratio which allows automated analysis. A minor drawback is a change in tissue size during processing which is largely reverted with appropriate mounting media, and needs to be considered when quantifying sizes or distances. We confirmed that crossing the BBB is performed by *C. neoformans,* and show for the first time in mice that microglia, the brain-resident macrophages, rapidly ingest traversed fungi, all within 24 hpi after systemic infection. This approach also provided new information on interactions of *C. neoformans* with murine hosts, including observation and quantification of titan cells *in situ*.

While we and others had previously detected cryptococci in upper airways of mice (42–44), we report for the first time that titan cells are abundant in airway turbinates of mice. Titan cells had been previously observed in nasal cavities of mammals with advanced symptomatic disease: from the necropsied fungal nasal mass of a dog with central nervous system (CNS) disease (45) and biopsy of nasal granuloma in a cat with localized disease (46). In our work, titan cells were found at high frequency in the first 24 hpi and at least up to 7 dpi, which indicates very strong titan-inducing or a titan-permissive environment in airway turbinates. This is the fastest known rate of titan cell formation, as titan cells are rare in the lung at 24 hpi but reach ~20% of fungi by 3 dpi (18). At this time, we cannot speculate on the specific airway conditions that induce or allow this abundance of titan cells. The presence of *Cryptococcus* in the upper respiratory tract is common in several animals, particularly dogs and cats (47), koalas (48, 49), and ferrets (50), and indicates either asymptomatic carriage or a symptomatic upper respiratory tract infection, which can progress to invasive infection (48). If the specific airway conditions allowing abundance of titan cells are conserved among mammals, and not a specific feature of *Mus musculus*, then titan cells would occur in airways of most mammals, and it is important to investigate their contribution to veterinary infection and disease.

We also note that veterinary observations in naturally exposed animals (45, 46) show fungal cells posterior (deep) into the upper airways, consistent with our observations in intranasally instilled animals. Similarly, penetration of nasal mucosa after intranasal instillation was reported before (44), demonstrating cryptococci have the capacity to overcome the first defensive layers in respiratory and olfactory mucosa and posits a tropism for the olfactory mucosa due to an unknown factor in this tissue. There are some anatomical differences between turbinates of animals and humans, a topic which was expertly reviewed by reference 51; immunological and/or tissue differences are the poorly characterized (52, 53). The consistent observations of significant fungal burden in upper airways, together with event of mucosal invasion in upper airways, suggest that the upper airway may be a relevant site of *C. neoformans* infection, at least in some animals, facilitating invasion of extra-airway sites either as a reservoir for fungal cells or as an additional site of access to the bloodstream via crossing of the nasal mucosa.

The data on nasal carriage of cryptococci in humans are sparse compared to other animals. Nevertheless, asymptomatic carriage of cryptococci is possible: one case report describes that after a pet ferret was diagnosed with cryptococcosis, its human owners showed positive cultures from nasal swabs, despite negative serum antigenemia. Thus, while colonization of human noses in immunocompetent individuals is possible, there are still insufficient data on the frequency of nasal carriage in healthy immunocompetent humans and whether any carriage is transient or long-lasting. Additionally, cryptococci can be detected in the upper respiratory tract of patients with symptomatic cryptococcal disease. One study in Lisbon, Portugal, observed abundant fungal cells in olfactory mucosa via histopathological analysis of the autopsies of patients who succumbed to AIDS-associated cryptococcosis (54), and a study in Nonthaburi, Thailand, recovered viable cryptococci from the nasopharynx of individuals recently diagnosed with AIDS-associated cryptococcal meningitis. While the presence of cryptococci in the nose may be attributable to the high fungal burden in these patients, it remains to be determined whether the presence of cryptococci in the upper airways of humans occurs during early disease of humans, and whether residency at this site contributes to pathogenesis and/or persistence in humans.

Bloodstream dissemination of cryptococci is widely accepted, as it is consistent with a diffuse and broad distribution of *C. neoformans* throughout the brain, in close proximity to blood vessels, observed in human post-mortem brains (55, 56) and in murine models (34, 35, 57). Murine studies have also detected bloodborne cryptococci after intranasal inoculation (25). An open question is how *C. neoformans* travels from airways to reach the bloodstream. A previous study showed rapid trafficking of yeast and spore-derived yeast into murine lung-draining lymph nodes, as early as 24 hpi (25, 31), and posited that escape from the lung into lymph nodes and then into the bloodstream provided a route to the murine brain. In that study, spores had quicker dissemination kinetics than yeast particles (25), via mechanisms yet unknown. It is also possible that direct traversal of lung alveoli may be an alternative route toward bloodstream dissemination: others have shown cryptococci can adhere to human lung epithelial A549 immortalized cells (9, 28) and other airway immortalized cell lines (29). This would be in line with our observation that cryptococci can penetrate epithelial layers, as we observed fungi penetrating the upper airway epithelium into the submucosa. However, our data are not conclusive regarding direct alveoli crossing by cryptococci. Strategies to escape from the upper and lower airways, contributions of different fungal particles and morphotypes (25, 58), as well as the relative contributions of lymphatic dissemination via direct angioinvasion into the circulating bloodstream remain to be determined.

Here, we observed free cryptococci in large blood vessels of lungs after intranasal infection. This is in line with work by others using systemic infections. Imaging of zebrafish embryos showed bloodstream had predominantly free cryptococci (59, 60). Flow cytometry and imaging of mice brains at 24 hpi showed cryptococci associated with brain endothelium cells, without the presence of phagocytes (27). Furthermore, *in vitro* work showed that free yeast traversal across endothelial cell layers was more efficient than THP-1 monocyte Trojan-horse traversal (26). Together, these works support the notion that free yeast are the predominant form in the bloodstream in mouse infections. Trojan-horse and free yeast traversal are not mutually exclusive, and free yeast in bloodstream would be compatible with Trojan-horse transit, if Trojan-horse transit was transient or specific to certain tissues. The relative ratio of traversal mechanisms and molecular mechanisms between mechanisms largely remains to be determined.

Rapid traversal of the BBB by *C. neoformans* was previously detected via intravital imaging to show that tail vein injection of particles of a certain size, such as fungi and inert polystyrene beads, leads to passive trapping of particles in small brain capillaries. Live fungi, but not killed fungi nor beads, traversed capillaries into the brain parenchyma 6 h after injection (7, 8, 61), indicating an active process of crossing by the pathogenic fungus. We also observed rapid crossing of the BBB by *C. neoformans* followed by close interactions with Iba1^+^ microglia, including ingestion of cryptococci, and the presence of fungal clusters, suggesting replication and growth within microglia or in the brain parenchyma. Our observations are in line with previous work determining that the brain niche is favorable to cryptococci growth, due to a combination of localized immune features and to favorable nutrition, such as an abundance of mannitol (12). We extend these results by showing early ingestion of fungi by Iba1^+^ cells in the very first day of infection, after endothelial crossing into the brain parenchyma. Rapid association of cryptococci with brain-resident macrophages is in line with recent publications reporting (i) ingestion of a small percentage of cryptococci by microglia 4 days post infection in the developing brains of zebrafish larvae (59, 60); (ii) cryptococcal association with phagocytes in mouse brains 7 days after intravenous infection, albeit a significant percentage of fungi are extracellular, fungi-containing cells were either microglia or phagocytes infiltrating from periphery (36); (iii) perivascular yeast in the BBB, either in free form or associated with phagocytic cells, 3 to 7 days after retro-orbital or tail vein inoculation (35). In contrast to these findings, two other studies find a significant percentage of fungi in mouse brains are extracellular. Up to 18 h after intravenous infection, the majority of yeast in brain lysates are extracellular, with fewer than 10% associated with leukocytes (10, 62). We posit that this discrepancy arises due to technical constraints: protocols that disrupt tissue may disrupt or discard clusters formed by microglia and fungi. In these cases, our data using intact tissues are likely more reflective of true *in vivo* interactions. The percentage of phagocyte-associated vs free fungi may vary during the course of infection; however, soon after crossing the BBB, the majority of cryptococci are interacting with and are in close proximity to microglia and trigger localized responses in adjacent microglia cells. Recent work showed that microglia were not effective fungicidal cells and, for some cryptococcal strains, can facilitate growth (36); these observations, together with ours, suggest that the fungal clusters associated with microglia at 24 hpi are the result of fungal growth facilitated by microglia association. This growth can be facilitated by increased copper levels inside microglia compared to extracellular brain parenchyma (36) and perhaps by the acidic pH of phagosomes which favors fungal growth compared to extracellular pH (reviewed in reference 63]). Our studies pave the way to study localized, spatially resolved host-fungal interactions underpinning invasion, such as determining the relative contribution of Trojan-horse traversal (9, 26, 30, 62), the contributions of Mpr1 (11), and interactions between hyaluronic acid in capsule and CD44 in endothelial cells to brain tissue invasion (64).

We observed apparent associations between multiple amoeboid microglia and clustered cryptococci. Expression of an amoeboid morphology is commonly associated with the inflammatory activation of microglia in several pathological processes (24). This immune activation likely occurs at all stages of infection, as was previously observed in a model of late cryptococcal meningitis, following intracerebral infection of mice (57). Amoeboid microglia were also observed after *Streptococcus pneumoniae* infection (65). In contrast, ramified microglia are still observed in the first hours after *Toxoplasma gondii* infection (66), demonstrating an interplay between neuro-immune responses and invading microbes. At this stage, there is no detectable recruitment of circulating monocytes to infection sites. Further characterization is needed to determine the functionality of these Iba1^+^ cells. While Iba1 is a well-accepted microglia marker, immune populations are now recognized as more complex and heterogeneous even within the same organ. Recently, transcriptomic and developmental profiles showed Iba1^+^ cells in brain are “true” microglia, parenchyma-resident macrophages that can migrate to vessels in response to invading stimuli, but a second population of Iba1^+^ cells are brain border-associated macrophages, and these subsets have subtle but important functional distinctions (23, 24).

One noteworthy observation from us and others is that the brain, seemingly well-protected by the multi-layered BBB, is effectively colonized by *C. neoformans*. Yet, its well-established sites with permeable capillary beds, such as the liver and the spleen, show a reduced burden of *C. neoformans. C. neoformans* trapped in liver sinusoids after intravenous injection was ingested by Kupfer cells, the liver-resident macrophages, and fungal burden was controlled in the first few hours post-injection (61). This was also demonstrated by the longitudinal imaging of bioluminescent fungi coupled to micro-computed tomography, up to 7 days after systemic and 4.5 weeks after intranasal infections (42). The corollary of these observations is that tissue barriers, including BBB, are not fully impermeable to pathogens, and that bloodstream permeability is not a major determinant of tropism over the course of infection, at least for *C. neoformans*. Instead, after the seeding of fungal pathogen in several organs, cryptococcal tissue tropism is likely most determined by the underlying tissue-specific immunity and by the pathogen’s adaptations to the specific nutritional conditions of the tissue, reminiscent of the “seed and soil” hypothesis by Paget (67).

Overall, we show here a high-content, high-resolution method to study fungal-host pathogens interactions, including fungal morphological analysis and tissue-immune interactions. This method potentiated several observations: we confirmed the presence of abundant titan cells in airway turbinates of mice, as we reported previously (6), we observed for the first time *C. neoformans* in the lamina propria of murine turbinates, and we confirmed the presence of cryptococci in the bloodstream of mice. Furthermore, we showed that in the early stages of brain invasion, similar to what occurs in lungs, *C. neoformans* associates rapidly with tissue-resident phagocytes, in this case, Iba1^+^ cells. Our work unveils early events in *C. neoformans* invasion of mammals and new insights into mechanisms of cryptococcal disease.

## MATERIALS AND METHODS

### Fungal strains

We used *C. neoformans* H99E, originating from JE Lodge laboratory and deposited into Fungal Genetics Stock Center, for the majority of experiments. Strains *ste50*Δ and *cac1*Δ were obtained from deletion library, created by the Madhani laboratory (68), through Fungal Genetics Stock Center. Strain H99-mCardinal (CnLT0004) was a gift from Edward Wallace and Laura Tuck; mCardinal is derived from KN99α to express the mCardinal red fluorescent protein (69), codon-optimized for *Cryptococcus*, and integrated into genomic safe haven 4 (70, 71) with RPL10/CNAG_03739 promoter and terminator, and a NAT resistance cassette. Cryptococci were grown from frozen 10% glycerol stocks on yeast extract-peptone-dextrose (YPD) agar plates for 2 days at room temperature, followed by culturing overnight at 37°C, 180 rpm, in YPD broth. Cryptococcal cell suspensions were counted in hemocytometer and diluted to the appropriate density.

### Mouse infections

C57BL/6J male mice, aged 8 to 12 weeks, were purchased from Charles River Laboratories, UK, and infected with 5 × 10^5^ CFU, unless otherwise specified, in sterile phosphate buffered saline (PBS, Oxoid, BR0014G). Intranasal infections were performed by placing 25 µL of yeast suspension into the mouse nares under isoflurane anesthesia. Intravenous infection was performed with 5 × 10^5^ CFU in 100 µL via tail vein injection. Mice were monitored every 6 h for the first 24 h, and then daily, for deterioration in health. We also imaged noninfected (sentinel) mice for tissue morphology, immunolabel, and dye-staining controls, including for CFW and antibody staining specificity. CX3Cr1^GFP/+^ mice were obtained from University of Exeter colony, a kind gift from Jon Witton and Peter C. Cook.

### Tissue extraction and fixation

Mice were culled via cervical dislocation. Skin was removed, and skull and thorax were opened to remove lungs. For samples intended for skull imaging, the skin, lower jaw, tongue, and attached skull muscles were removed. When needed, cardiac bleeds were performed under isoflurane anesthesia. All tissues were fixed for 48 h in approximately 20-fold volume of 4% formaldehyde at room temperature with gentle agitation in a rotating shaker. Tissues were then rinsed several times in PBS containing 0.02% azide and stored at 4°C until further processing. Unless otherwise noted, 0.02% azide was added to all PBS-based solutions to prevent microbial growth.

### Decolorization and decalcification

After fixation, skulls were placed in decolorization solution made with 30% dilution of CUBIC reagent 1, as in reference 14, in 0.1M PBS [CUBIC reagent 1 was prepared with 25 wt% urea (Thermo Scientific, U/0500/65), 25 wt% N,N,N′,N′-tetrakis (2-hydroxypropyl)ethylenediamine (Thermo Scientific, L16280.AE) and 15 wt% Triton X-100]. Skulls were incubated at 37°C for 48 h in 5 mL decolorization solution with the solution being refreshed at least four times until it remained clear. Samples were washed twice in 5 mL PBS and placed in 40 mL decalcification solution (0.2 M EDTA in 0.1M PBS adjusted to pH 8–9 with sodium hydroxide) for 72 h at 37°C. Skulls were washed twice in 5 mL PBS and kept at 4°C in PBS until further processing. Lungs were decolorized only for Fig. 5, but not decalcified.

### Slicing and tissue clearing

Whole organs were submerged in up to 5 mL X-CLARITY hydrogel monomer with initiator solution. Oxygen was removed from the solution via degassing with nitrogen flow prior to organ submersion and again after submersion. Tissues were incubated at 4°C overnight followed by 3 h at 37°C, with gentle agitation. Tissues were washed in 5 mL PBS to remove hydrogel. Organ sections were obtained by 300 or 400 µm sagittal cuts with a vibratome. In some cases, tissues were cut before embedding in hydrogel, but we found tissue to become more stable if hydrogel-embedded was performed before cutting. All tissues were cleared with X-CLARITY following manufacturer instructions. Tissues were incubated in 2 mL X-CLARITY tissue-clearing solution at 37°C overnight. Tissues were then cleared in an electrophoretic tissue clearing chamber (ECT, LogosBio instruments) with a current of 1.5A, circulation speed of 30 rpm, at 37°C for 3 h, and inverted halfway through incubation. Samples were washed twice in 5 mL PBS and stored at 4°C in PBS, until further analysis.

### Staining

Organ sections were stained with CFW for 48 h with gentle rotation at room temperature prior to blocking and staining with antibodies and dyes. Tissues were blocked overnight in 1 mL Fc block solution, containing 5 µg/mL 3.G2 Fc-block (BD Bioscience, 553142), 3% bovine serum albumin (BSA), 0.02% azide, and 0.1% Triton X-100 in PBS. Tissues were stained with antibodies and dyes listed in Table 1, in Fc blocking solution, for 48 h with gentle agitation. After staining, samples were rinsed in 1 mL PBS overnight at room temperature with gentle agitation. Prior to imaging, tissues were mounted in refractive index match solution (80% glycerol in water).

**TABLE 1:** List of antibodies and dyes used

Antigen-fluorophore	Clone	Catalog #	Company	Staining concn (dilution from supplier stock if concn not available)	Target
CD31-PE	MEC13.3	102507	Biolegend	5–7 µg/mL	Endothelium
CD31-AF647	MEC13.3	102515	Biolegend	5–15 µg/mL	Endothelium
Podocalyxin-PE	10B9	107930	Biolegend	5–7 µg/mL	Endothelium
AF647 – Podocalyxin	192703	FAB1556R	Bio-techne	22.5 µg/mL	Endothelium
Iba1-AF647	EPR16588	AB225261-1001	Abcam	5 µg/mL	Iba1, microglia specific marker
EpCAM-PE	G8.8	15228839	Invitrogen	5 µg/mL	Lung epithelial
Helix NP Blue		425305	Biolegend	25 µM	Nuclei
Helix NP Green		425303	Biolegend	25–200 µM	Nuclei
Propidium iodide		P4864	Sigma	20 ug/mL	Nuclei
CFW (Fluorescent Brightener 28)		F3543	Sigma	50–100 µg/mL	Chitin in cell wall of fungi

### Imaging and processing

For imaging tissue sections, we used two confocal microscopy systems (Table 2). On a Zeiss LSM 880 Airyscan, we firstly generated a tissue outline by briefly imaging with a 10× objective (PApo 10 × 0.45 – dry), which optimized the imaging area for subsequent imaging in detail with a 25× LD LCI PApo 25×/0.8 Imm Corr D objective, with oil immersion (RI = 1.518). Combinations of 405 nm, 488 nm, 561 nm, and 633 nm lasers were used, depending on antibody/dye combinations. In most cases, the visible beam path was set up to use a tri-main beam splitter (488/561/633) and the UV path used a-405, beam splitter. At minimum, images were acquired with a zoom of 0.8 and a pixel voxel of 0.83 µm × 0.83 µm × 9 µm with resolution increased in some regions of interest (ROI) to >0.531 µm × 0.531 µm × 6 µm. Multicolor images were obtained using a combination of spectral 32 channel GaAsP photomultiplier tubes (PMT) and multi-alkali PMTs detectors. Antibody staining controls were performed on unstained and single-stained samples, and informed acquisition and processing of images (see Fig. S1 and S4). Zeiss Zen (Blue edition) 2.3 or (Black edition) was used for stitching images together for full maps, with a 15%–20% overlap.

**TABLE 2:** Imaging systems used

Figure	Imaging system
Fig. 1	Airyscan
Fig. 2	Airyscan
Fig. 3	Airyscan
Fig. 4	Airyscan
Fig. 5	DragonFly
Fig. 6	Airyscan
Fig. 7	Airyscan
Fig. S1	Airyscan
Fig. S2	Airyscan
Fig. S3 Fig. S3E	Deltavision ELITE Dragonfly
Fig. S4	Airyscan
Fig. S5	Airyscan
Fig. S6	Dragonfly
Fig. S7	Airyscan
Fig. S8	Airyscan

Some sections were imaged on a Nikon Ti2 microscope body (Nikon Microsystems) with a DragonFly505 40 µm pinhole spinning disk microscope (Andor, Belfast) operating Fusion software. UV fluorophores was excited with a 405 nm laser and the emission collected through a Semrock TR-DFLY-F445-046 filter. Green fluorophores were excited with a 488 nm laser and the emission collected through a Semrock TR-DFLY-F521-038 filter. Full configuration of the microscope is available at https://www.fpbase.org/microscope/ZR9nEdoko3bwnnU66QAJUE/. Fluorescence was collected on an Andor Sona sCMOS camera with 2 × 2 binning for large area maps with some ROI imaged with 1 × 1 binning. Overview skull maps were generated using a Nikon 4×/0.2NA PlanApo lambda lens, while higher-resolution 3D acquisitions were acquired using a Nikon 20×/0.75NA PlanApo lambda lens. Images were stitched using 5% overlap on Fusion ClearView software. Z-stacks and step size are indicated in the figure legends.

### Automated measurement of fungal cell diameter

Automated 2D fungal cell segmentation was achieved by selection of an ROI, and z-stacks were transferred to ImageJ (72), the fungal cell wall CFW channel was extracted and normalized. Areas of certain images with high background autofluorescence (bone protrusion in nasal cavity) were cropped out prior to analysis. Loss of signal intensity with depth of tissue was corrected with xyz normalization through z-stacks in ImageJ. Manual boundary tracings were performed on z-stacks (before sum projection), using Fiji straight or freehand line tool for cross-section and object boundary tracing for interpolated diameters based on cell area. For StarDist, ROI were sum projected to produce a 2D image from the 3D z-stack, and 2D images analyzed with StarDist (73), with probability/score threshold of 0.15, an overlap threshold of 0.40, and a boundary exclusion of 5 for single-cell area analysis. Cell diameters were then computed from the area of objects, using the formula for area of a circle = π × radius^2^.

### Data analysis

Images were extracted and figures assembled using a combination of Zeiss Zen Blue edition 2.3, Zen Black (Carl Zeiss Microscopy), Imaris (Oxford Instruments), and ImageJ (NIH, USA). Unless stated on figure legend, no image processing steps were performed. Distances of cryptococci to blood vessels were measured in 3D stacks, using manual tracing. Areas of microglia and fungi were measured using spatial stereology, which maps areas with minimal bias based on methods by references (74, 75). Graph Pad Prism was used for graphs and statistics, Biorender was used to create diagrams.

## SUPPLEMENTAL MATERIAL

Supplemental figures mbio.03078-23-s0001.pdf Fig. S1-S8.

Video S1 mbio.03078-23-s0002.mp4 Illustration of infected skulls related to Fig. 1.

Video S2 mbio.03078-23-s0003.mp4 *C. neoformans* in turbinates.

Video S3a2 mbio.03078-23-s0004.wmv *C. neoformans* ingested and associated with Iba1 microglia.

Video S3b mbio.03078-23-s0005.mp4 *C. neoformans* ingested and associated with Iba1 microglia.

Video S3c mbio.03078-23-s0006.wmv *C. neoformans* ingested and associated with Iba1 microglia.

Video S3d mbio.03078-23-s0007.wmv *C. neoformans* ingested and associated with Iba1 microglia.

Supplemental Results and Methods mbio.03078-23-s0008.docx Additional details regarding clarification of tissues.

ASM does not own the copyrights to Supplemental Material that may be linked to, or accessed through, an article. The authors have granted ASM a non-exclusive, world-wide license to publish the Supplemental Material files. Please contact the corresponding author directly for reuse.
