# Supplementary material for: Cryptococcus neoformans rapidly invades the murine brain by sequential breaching of airway and endothelial tissues barriers, followed by engulfment by microglia
Source: mBio. 2024 Mar 21;15(4):e03078-23. doi: 10.1128/mbio.03078-23 (PMC11005363; doi:10.1128/mbio.03078-23)
Supplement: Supplemental figures — Fig. S1-S8. [file mbio.03078-23-s0001.pdf]

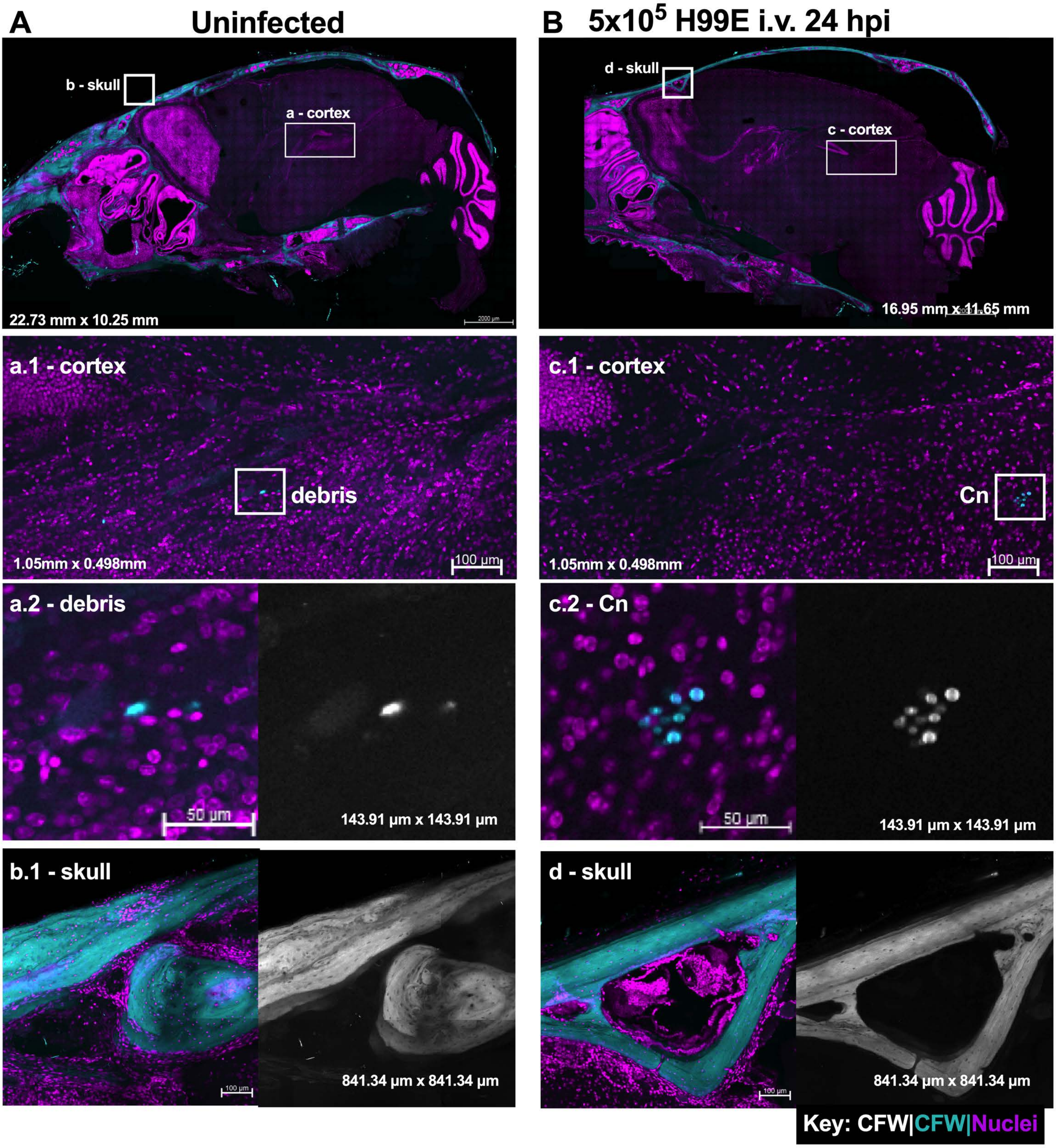

SFig.1. Reliable identification of *C. neoformans* based on morphology and specific dye staining. Skulls from **A)** uninfected and **B)** 5x10^5 H99E i.v infected C57bl/6 male 24 hpi. Consecutive magnifications of regions of interest (a→a.1→a.2; b→b.1→b.2) allowed identification of *C. neoformans* with a high degree of confidence by their characteristic morphology (circular yeast of variable size with narrow base budding) separating them from CFW debris. In b.1 and d.1, bone shows unspecific CFW labelling, but is clearly distinguishable from the pattern of staining observed for *C. neoformans* cells.
All images are maximum intensity projections of **A)** x4 z-step 228 µm deep, **B)** x4 z-step 225 µm deep. Skulls stained and imaged in parallel using the same imaging and display settings with 50 µg/ml CFW (cyan or white) for cell wall of *C. neoformans* and 40 µg/ml PI (magenta) for DNA. Scale bar shown for each panel.

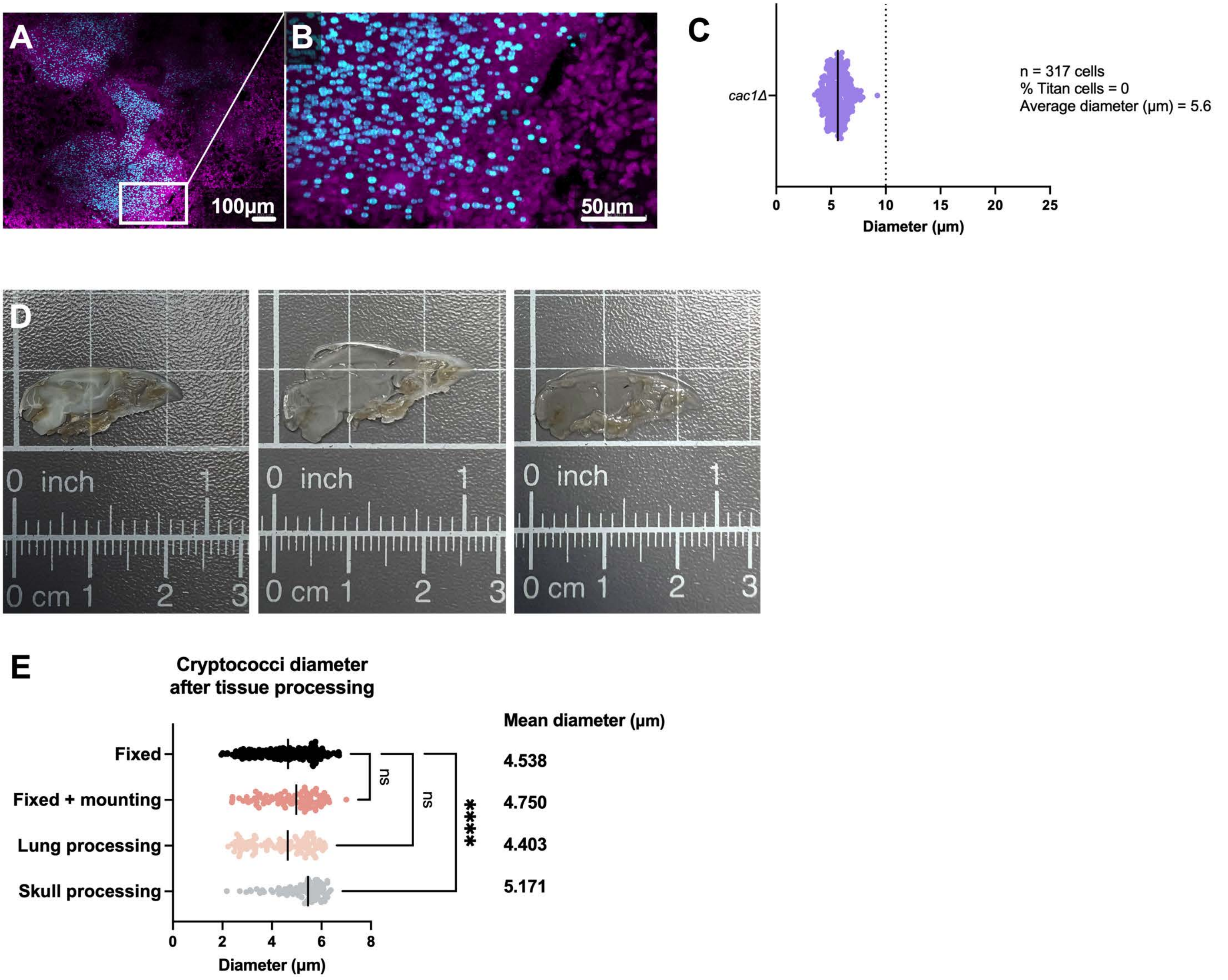

SFig 2. Pipeline to detect cryptococcal cell size distribution in infected tissues. (related to Fig.2)
Map of infected lung showing CFW-stained cryptococcal cells and PI-stained nuclei of lung cells, with consecutive magnifications from tissue to subcellular resolution. Shown are several steps in image processing and analysis which allowed measurements of fungal cell morphology *in situ*. **A-C)** To demonstrate the protocol is sensitive to changes in fungal size, hypo-titan strain (*CNAG_03202 cac1Δ*) was imaged at 24hpi, conditions where titan cells are exceedingly rare. We confirmed titan cells were not detected in these conditions, and **A)** Section of *cac1Δ*-infected lung. **B)** Region of Interest (ROI) used for analysis. **C)** Fungal diameter via StarDist. **D)** Skull expansion after processing and shrinkage in RI-matched mounting media. **E)** Lung processing does not change fungal size, however skull processing requires decalcification and decoloring steps and these processes result in 1.13 fold-increase in fungal diameter from 4.5 µm to 5.2 µm.
Images **(A-C)** from lung of C57bl/6J male mice, infected with 5x10^{7} *cac1Δ*, 1 dpi. Lungs stained with PI (magenta) and CFW (cyan). Images correspond to **A-C)** Extended depth of field image, 132 µm depth, 67x 2µm z-steps. **B)** 132 µm depth, 67x 2µm z-steps. Scale bar in images. Graph in **E** shows average (bars) and 86-120 yeast measured in each group; p-value calculated via one-way ANOVA, ns, p>0.05 , ****,<0.0001.

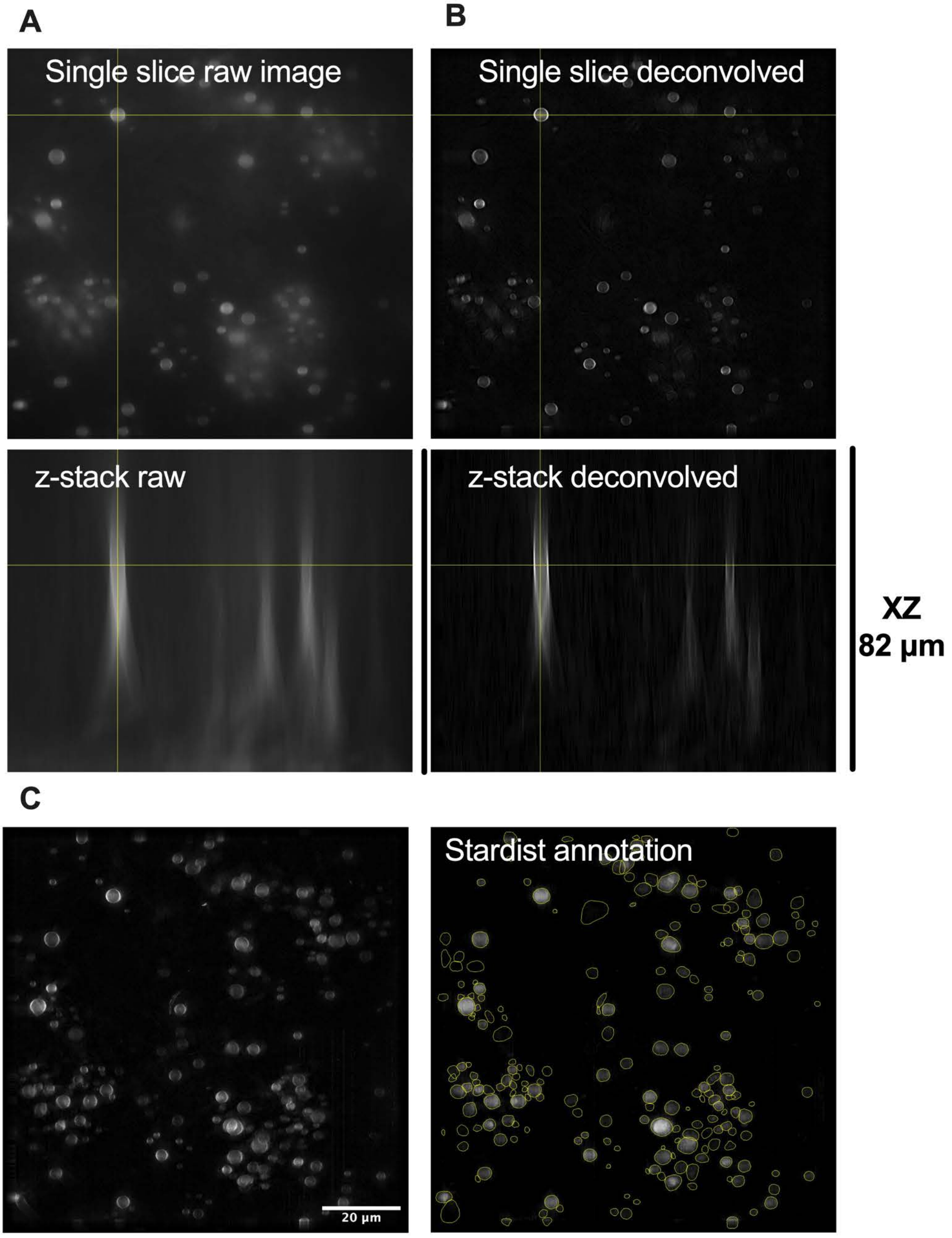

SFig 3. Clarification of tissues allows increased depth of imaging in non-confocal microscopes, which can be harnessed to measure fungal morphology and size in thick slices.
A clarified lung was imaged up to 176 µm depth, with 2 µm z-steps in a widefield microscope Deltavision ELITE (see methods for full information). After deconvolving, removing out of focus and low intensity top and bottom z-steps, stacks of usable quality could be obtained between z-steps 12 to 53, for a final stack totalling 82 µm (first11 z-steps are used for deconvolution calculations and last z-steps had lower intensity of signal).
A) 1 single z-stack pre-deconvolution and **B)** post deconvolution, **C)** z-projection of stacks 12-53, showing fungal morphology. Lung from C57bl/6J male animal, infected with for 7 days. Scale bars in image.

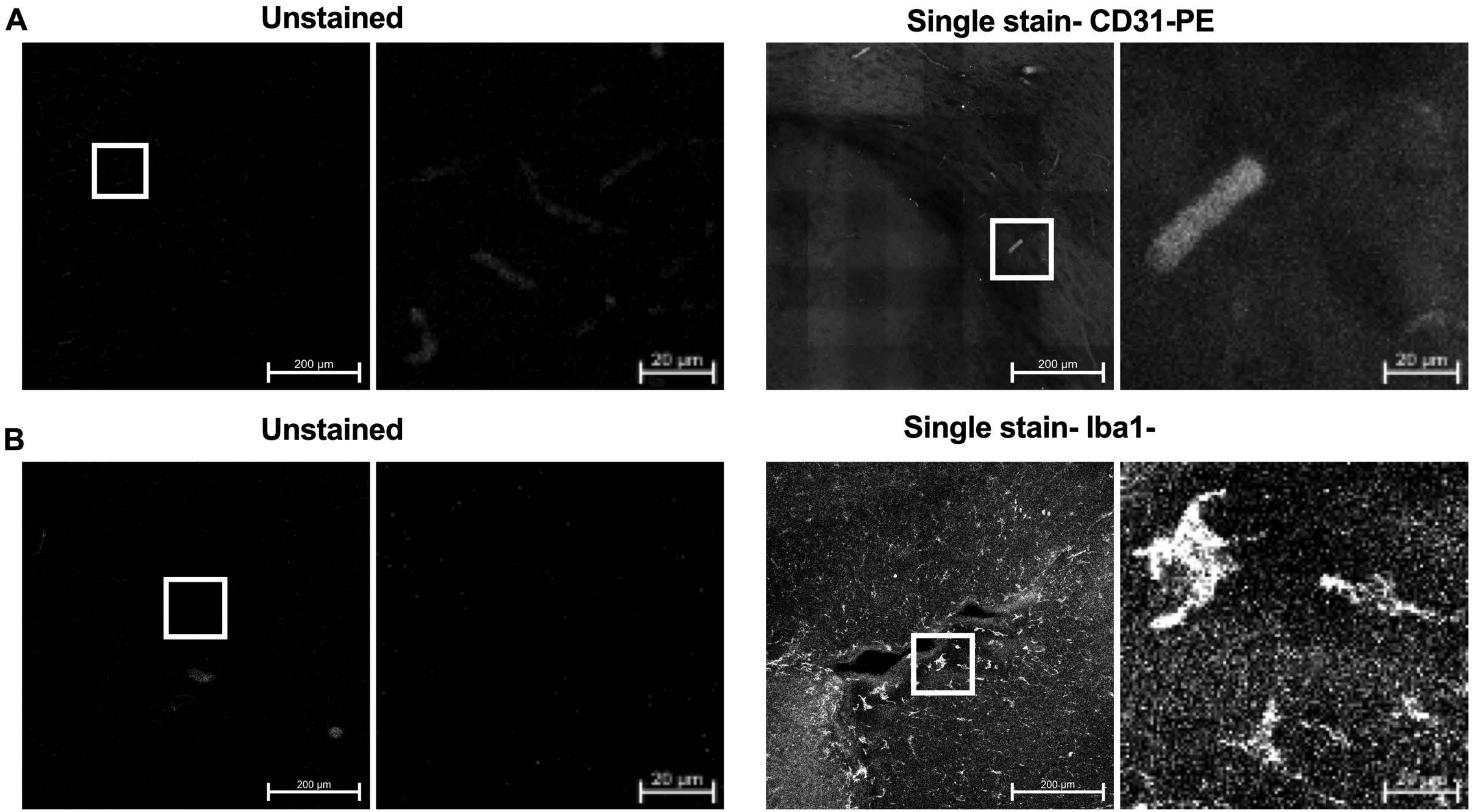

SFig4. Staining controls for antibodies, showing specific staining of **A)** CD31 and **B)** Iba1 antibodies. See also SFig.1 for CFW and nuclear dye controls. Similar data for CD31+Pdx (data not shown). All images are maximum intensity projections. Scale bar in images.

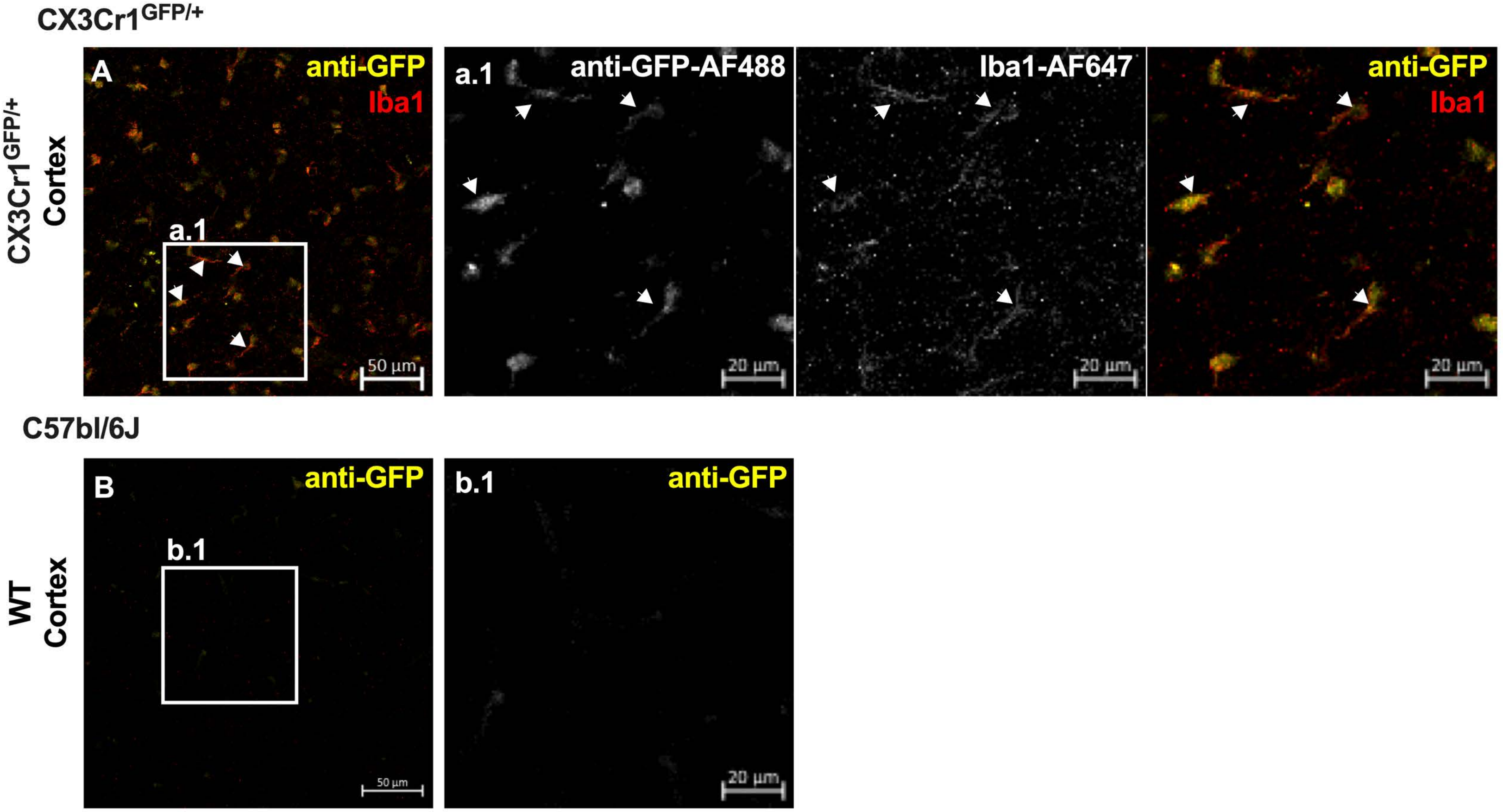

SFig5. CX3CR1-GFP in brain colocalized with Iba1, showing little peripheral monocyte infiltration. Co-expression of CX3Cr1 and Iba1 in brain parenchyma confirms these are microglial cells. Recently recruited peripheral monocytes are identified by CX3Cr1^{+}, Iba1^{-}. Detection of no recent inflammatory infiltrate indicates little to no peripheral infiltrate at this stage of infection. Lack of staining in WT mice indicates specificity of anti-GFP (GFP-booster, Abcam) mAb in detecting CX3CR1-GFP^{+} cells. A) Brain parenchyma of CX3Cr1$^{GFP/+}$ mice, labelled with anti-GFP-AF488 (GFP-booster, Abcam) and Iba1-AF647, magnified in a.1. B) WT C57Bl/6J mouse, lacking CX3CR1-GFP transgene, was stained with anti-GFP, magnified in b.1. Images from 24h post-IV infections of 5x10^{5} CFU of H99-mCardinal (A) and H99 (B). Arrows indicate examples of CX3Cr1$^{GFP/+}$ (yellow) and Iba1 (red) co-localization. Images are maximum intensity projections with 202 µm depth (1 µm x 203 z-steps). Scale bar in images.

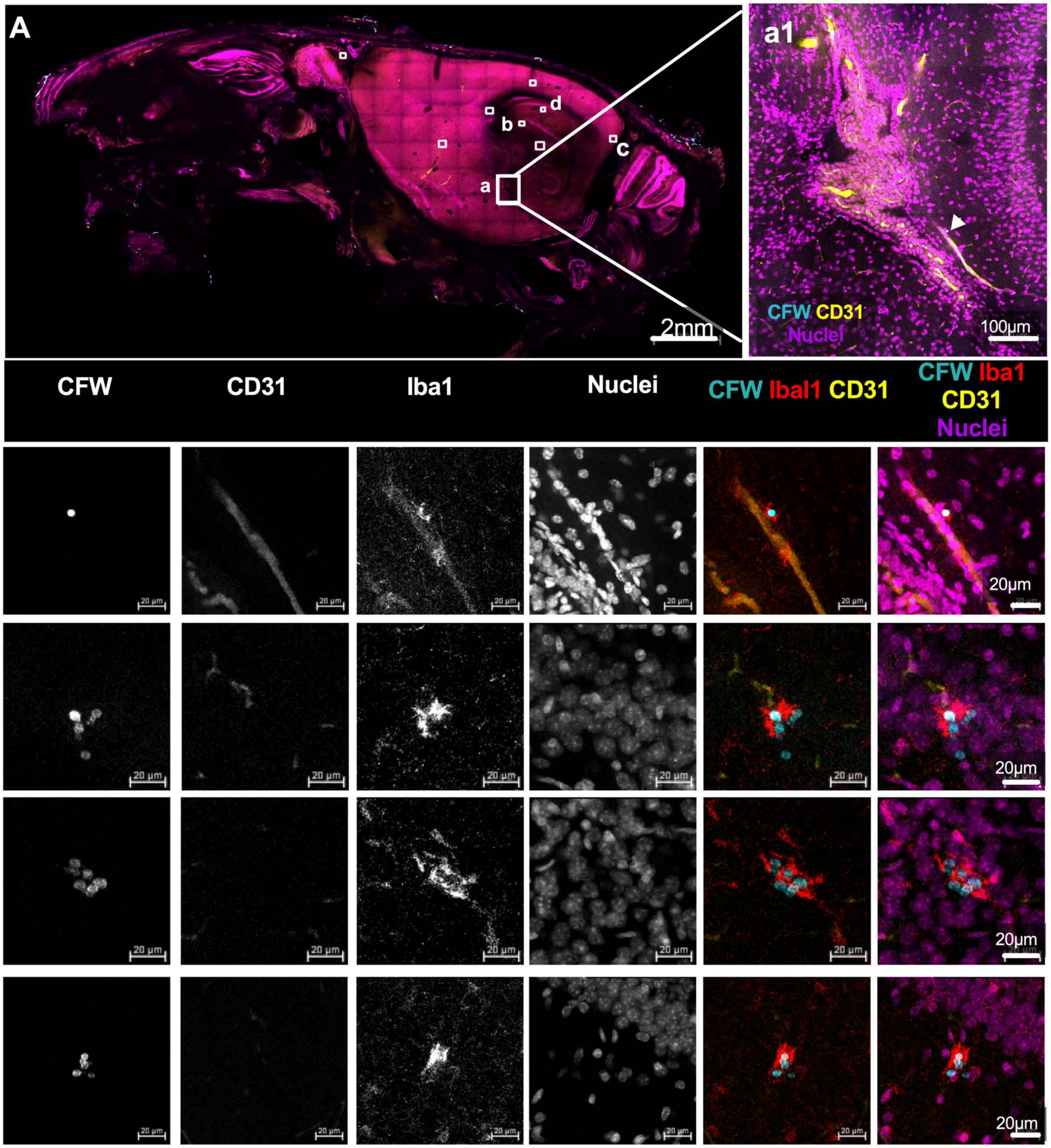

SFig. 6. *C. neoformans,* 24h after intravenous infection, is mostly associated with Iba1$^+$ cells. (related to Fig. 7)

Mouse skulls stained to study localization of fungal cells in relation to blood vessels (CD31$^+$) and association with Iba1$^+$ cells (marker for brain resident microglia, including border-associated microglia). Representative images are shown, with 12 fungal events (9 clusters and 3 single cells); of those 6 clusters and 2 single cells were associated with Iba1$^+$ cells, with **a2** localized, outside and in close proximity to CD31+ endothelium. Images from **A)** Skull map displaying the 9 locations, C57bl/6J male, 24hpi after 5x10^5 CFU of H99E infection i.v.. **a1)** Single *C. neoformans* cell (white arrow) was located outside of the blood vessel, localized near the lateral ventricle area. **a2-d)** Maximum projection of **a1)** 18 µm (2x9 µm z-steps; **a2)** 135 µm (4x45 µm z-steps); **b)** 54 µm (7x 9 µm z-steps); **c)** 45 µm (6x 9 µm z-steps); **d)** 63 µm (8x 9 µm z-steps). Sagittal slice corresponds to Allen Brain map slices 6-11. Scale bars in images.

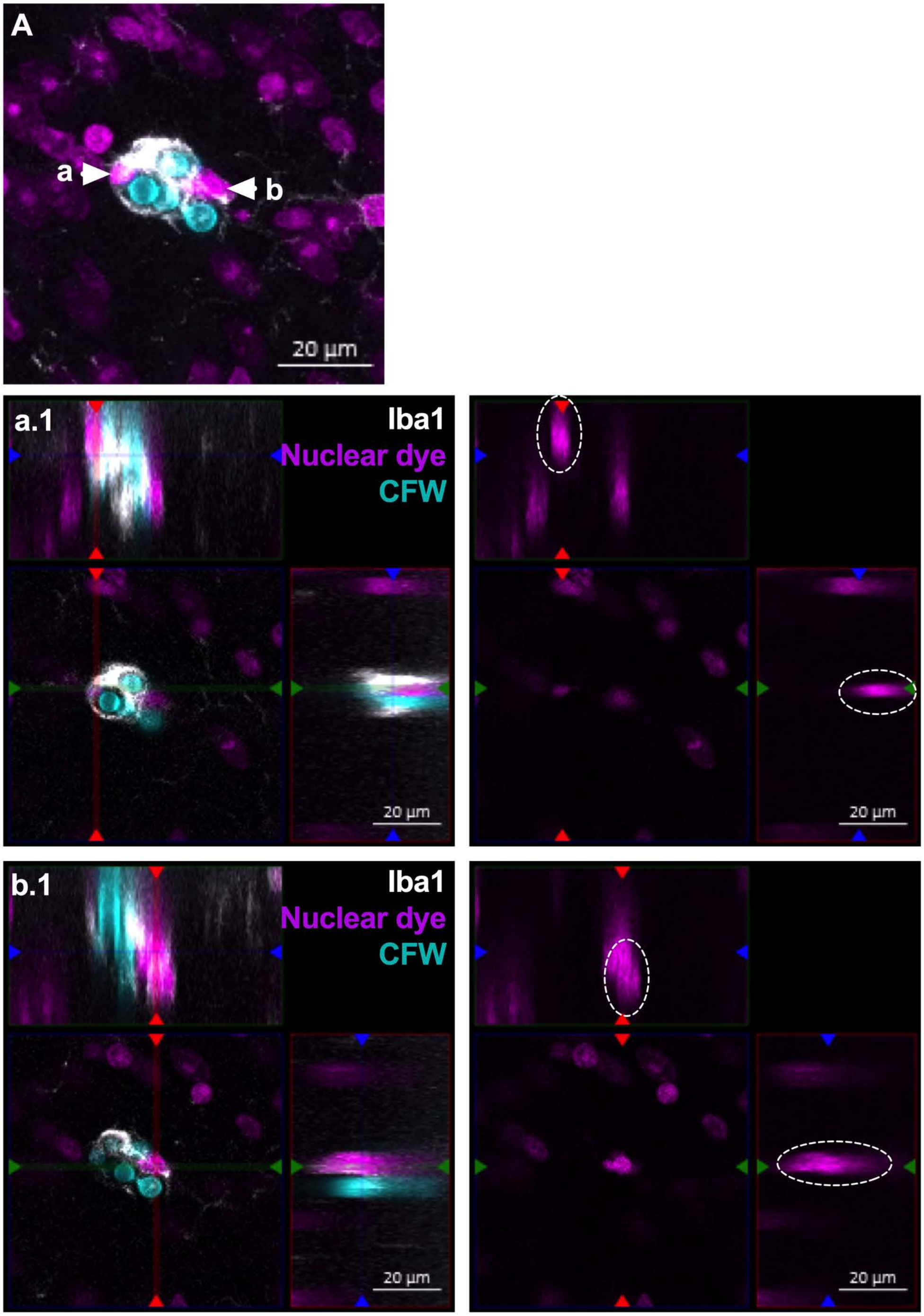

SFig. 7. *C. neoformans*, 24h after intravenous infection, is mostly associated with multiple or fused Iba1+ cells. (related to Fig.7)
Representative images of cryptococci clusters associated with $Iba1^+$ cells, as shown by multiple nuclei (magenta). Images are xyz projections, from panel b1 in Figure 7. Scale bars in images.

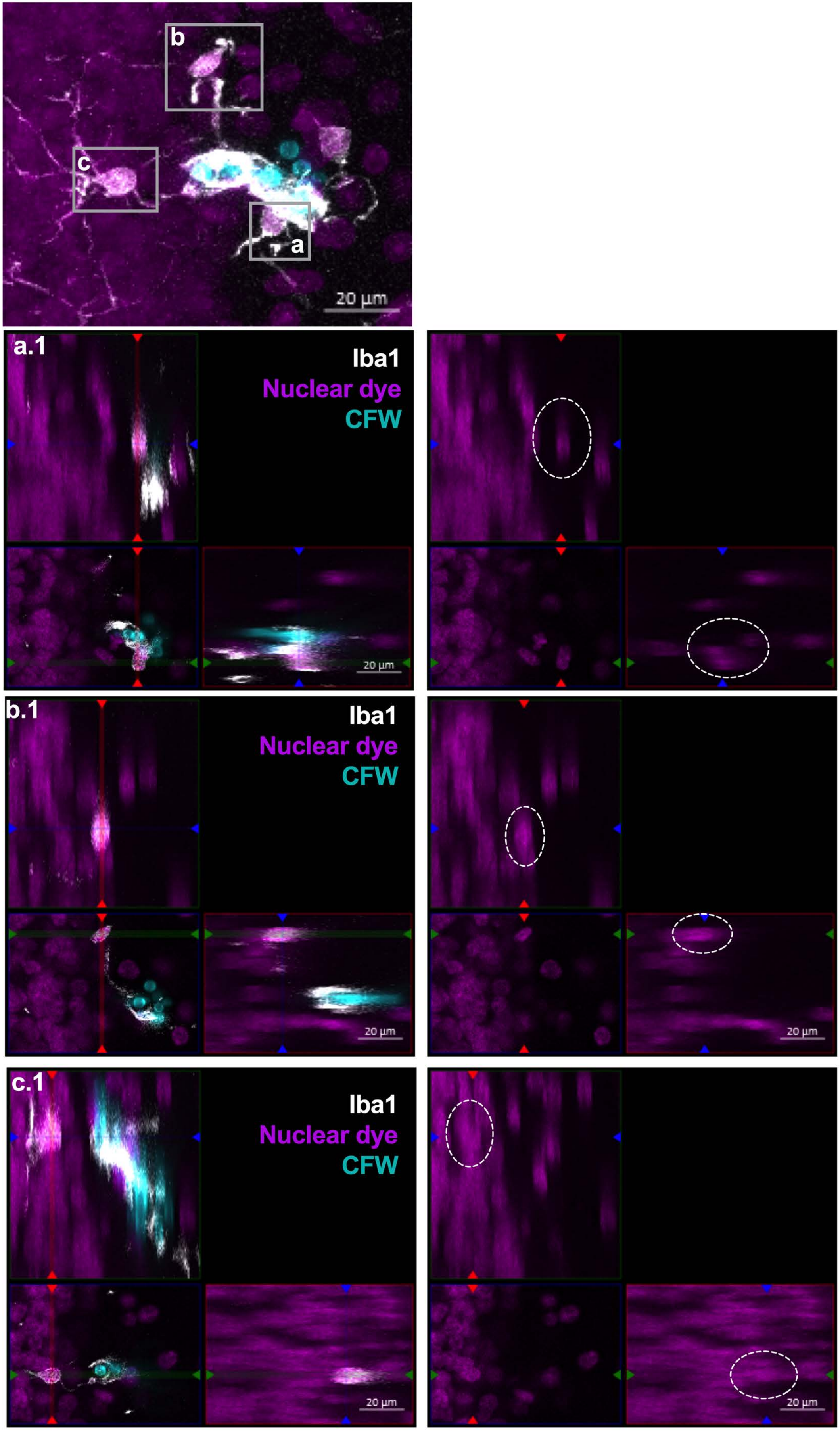

SFig. 8. *C. neoformans*, 24h after intravenous infection, is mostly associated with multiple or fused Iba1+ cells. (related to Fig.7)

Representative images of cryptococci clusters associated with Iba1$^+$ cells, as shown by multiple nuclei (magenta) and communication with neighboring microglia. Images are xyz projections, from panel c1 in Figure 7. Scale bars in images.
