# Supplementary material for: Cryptococcus neoformans rapidly invades the murine brain by sequential breaching of airway and endothelial tissues barriers, followed by engulfment by microglia
Source: mBio. 2024 Mar 21;15(4):e03078-23. doi: 10.1128/mbio.03078-23 (PMC11005363; doi:10.1128/mbio.03078-23)
Supplement: Supplemental Results and Methods — Additional details regarding clarification of tissues. [file mbio.03078-23-s0008.docx]

**Supplemental Video Legends**

**Video S1:**

S Video 1: Video illustrating data visualization in infected skulls. Colors represent: Cyan-CFW, magenta-nuclei. Related to Fig.1

**Video S2**

**SVideo2.** *C. neoformans* in turbinates**.** Colors represent: Cyan-CFW, magenta-nuclei**.** Video is Crop-720p,30fps. Related to Fig 3a.

**Video S3 (a2 through d).** *C. neoformans* ingested and associated with Iba1 microglia. Colors represent: Cyan-CFW, red-Iba1, yellow-CD31+Pdx. Related to SFig.6 (a2 through d).

**Supplemental Results:**

**Automated pipelines for titan cell measurements in clarified tissue**

We tested several automated and manual pipelines to measure the number and size distribution of cryptococcal cells, including ImageJ (StarDist macro, open access software^72^). To ensure these pipelines were accurately measuring titan cells (Fig 2D-J), we measured fungal sizes with manual cross-section to ascertain diameter of titan cells (the most commonly used method ^76–78^ ). We noted that automated algorithms require calculation of diameter from the area determined by boundary tracing, and thus we also performed manual boundary tracing (interpolated ∅– cell area). Both boundary tracing methods (manual and StarDist) resulted in an increased diameter, and thus a higher % of titan cells, than cross-section diameter measurements, and this needs to be considered when comparing our data to previous studies. Still, we found that manual boundary tracing and automated measurements were comparable in detecting number, and average diameter of cryptococci (Fig.2I).

To examine variations in titan cell formation *in situ*, and the accuracy of our fungal size pipelines, we required tissues where titan cells were formed and tissues were titan cells were rare. From the available deletion library, we obtained the *cac1Δ* strain, which fails to produce titan cells *in vitro*^77,79^, and as control, we used *ste50Δ*, which having undergone similar genetic engineering retains wild-type virulence and capacity to form titan cells^13^. At 5 days post infection (dpi) with *ste50*Δ (5x10^7^ CFU), mouse lungs contained a wide size range of cryptococcal cells, with an average fungal cell diameter of 8.4 μm. To ensure we had and few titan cells and abundant fungi in lungs, we imaged *cac1Δ* at 24 hpi, as titan cells are rare in lungs at 24 hpi^18^, whilst ensuring *cac1Δ* fungi were abundant and not yet cleared by host immune system. In lungs infected with *C. neoformans* *cac1*Δ (5 x 10^7^ CFU) and imaged 24 hpi, our pipelines detected no titan cells and all cryptococci had a cell body size below 6 μm, with some cells as small as 2-3 μm (SFig.3).

X-CLARITY can expand some tissues significantly, but this expansion is largely reversed on mounting with a refractive index (RI) match solution, the last step before imaging ^17,80^ (SFig.2). To confirm our imaging pipeline was reliably detecting titan cells, we submitted YPD-grown cryptococci to our clarification protocols: skull processing requires fixation, decalcification and decolorization, clarification, staining, and mounting; lung processing requires fixation, clarification, staining and mounting. Fungal cell diameter was not affected by fixation; in certain conditions, diameter was 1.13-fold increased compared to fixed cells (SFig.2). We note to readers that we reported fungal diameter values measured in tissues, and applied no correction factor for this potential increase in fungal diameter. In Fig.3B, correcting the fungal diameter signifies mean fungal diameter is 9.97 μm, with 52% of cells being >10 μm (compared to 11.3 μm and 59% of titan cells), which is still a remarkable >50% of cryptococci in airways at 24 hpi classified as titan cells, and does not affect results any of our conclusions.

**Clarification allows increased depth of imaging in widefield microscopes**

At this point, we reasoned that clarification allows thick tissues to be imaged in other microscopes, allowing certain measurements to be made in a great range of microscopes. Thus, we imaged lung sections in a non-confocal microscope with deconvolution capabilities, such as widely available microscope Deltavision ELITE. A clarified infected lung was imaged up to 176 μm depth (SFig.3), with 2 μm z-steps. After deconvolving, removing out of focus and low intensity images (first eleven z-steps are used for deconvolution calculations and last z-steps had lower intensity of signal), images of usable quality, with enough resolution to observe fungal cells, could be obtained between z-steps 12 to 53, for a final stack totaling 82 μm depth, and an actual tissue penetration of ~100 μm, which greatly comparable to confocal microscopy. A separate experiment shows nuclear staining can also be observed in thick tissues after clarification (data not shown). Whilst the level of detail obtained does not reach confocal resolution, our data indicates tissue clarification expands the range of microscopes that can be used to image thick tissue and to image structures approximately the size of cryptococci or mammalian nuclei (>1-4 μm); but not sufficient for structures smaller than those.

**Supplemental methods:**

Tissue clarified as above was imaged on a widefield microscope (named Deltavision ELITE). Widefield 3D volumes of infected lung tissue were captured using an Olympus 20/0.45 NA CPlanFluorite LWD objective lens and a PCO Edge sCMOS camera (Photometrics, UK) mounted on a DeltaVision Elite Olympus IX73 microscope with Softworx Resolve 3D software (Image Solutions, UK). CFW fluorescence was excited using a Lumencore UV LED and a Semrock DAPI filter set for excitation (390/18 nm) and emission (435/48 nm) fluorescence. The 3D volume was optically sectioned at nyquist sampling rates of 0.2 µm followed by in-built SoftWorx deconvolution algorithm, using 20 cycles of aggressive ratio. Deconvolved images were visualized in FIJI using Stacks, followed by normalization in ImageJ and background subtraction before quantification.

For fungal cell size changes after tissue processing, fungi from a fresh overnight YPD culture were fixed for 30 min at room temperature and then, decolorization for 1h at 37 °C, decalcification for 1h at 37 °C, and tissue-clearing for 1 h at 37 °C (skull processing), using the same solutions as above. Clarification only processing was similar, except decalcification and decolorization was not performed (Clarification). Centrifugation of fungal cells and 2 washes with PBS were performed at every change. Fungi were then stained with 25 µg/ml CFW overnight and immersed into mounting media for 2h to mimic time required for imaging setup of whole tissues. Fungal cells were then suspended in PBS, and imaged immediately in a coverslip-slide using the Dragonfly microscope.

**References**

1. Dambuza IM, Drake T, Chapuis A, Zhou X, Correia J, Taylor-Smith L, LeGrave N, Rasmussen T, Fisher MC, Bicanic T, Harrison TS, Jaspars M, May RC, Brown GD, Yuecel R, MacCallum DM, Ballou ER. 2018. The Cryptococcus neoformans Titan cell is an inducible and regulated morphotype underlying pathogenesis. PLoS Pathog 14:e1006978. <https://doi.org/10.1371/journal.ppat.1006978>
2. Hommel B, Mukaremera L, Cordero RJB, Coelho C, Desjardins CA, Sturny-Leclère A, Janbon G, Perfect JR, Fraser JA, Casadevall A, Cuomo CA, Dromer F, Nielsen K, Alanio A. 2018. Titan cells formation in Cryptococcus neoformans is finely tuned by environmental conditions and modulated by positive and negative genetic regulators. PLOS Pathog 14:e1006982. <https://doi.org/10.1371/journal.ppat.1006982>
3. Trevijano-Contador N, Rossi SA, de Oliveira HC, Llorente I, Correia I, Pla J, Zaballos Á, Ariño J, Zaragoza O. 2017. Cryptococcus neoformans can form titan-like cells in vitro in response to multiple signals that require the activation of several transduction pathways. bioRxiv. <https://doi.org/10.1101/193540>
4. Gish SR, Maier EJ, Haynes BC, Santiago-Tirado FH, Srikanta DL, Ma CZ, Li LX, Williams M, Crouch EC, Khader SA, Brent MR, Doering TL. 2016. Computational analysis reveals a key regulator of cryptococcal virulence and determinant of host response. mBio 7:e00313-16. <https://doi.org/10.1128/mBio.00313-16>
5. Orlich M, Kiefer F. 2018. A qualitative comparison of ten tissue clearing techniques. Histol Histopathol 33:181–199. <https://doi.org/10.14670/HH-11-903>
